# Supplementary material for: Vpr counteracts the restriction of LAPTM5 to promote HIV-1 infection in macrophages
Source: Nat Commun. 2021 Jun 17;12:3691. doi: 10.1038/s41467-021-24087-8 (PMC8211709; doi:10.1038/s41467-021-24087-8)
Supplement: Supplementary file 1 — Supplementary Information [file 41467_2021_24087_MOESM1_ESM.pdf]

Supplementary Information

Vpr counteracts the restriction of LAPTM5 to promote HIV-1 infection in macrophages

Li Zhao[1,3], Shumei Wang[1,3], Meng Xu[2], Yang He[2], Xiaowei Zhang[1,3], Ying Xiong[1,3], Hong Sun[1,3], Haibo Ding[1,3] Wenqing Geng[1,3,4], Hong Shang[1,3,4,5], and Guoxin Liang[1,2,3,4,*]

[1]Key Laboratory of AIDS Immunology of Ministry of Health, Department of Laboratory Medicine, The First Affiliated Hospital, China Medical University, Shenyang, China

[2]Research Institute for Cancer Therapy, The First Affiliated Hospital, China Medical University, Shenyang, China

[3]National Clinical Research Center for Laboratory Medicine, The First Affiliated Hospital of China Medical University, Shenyang, China

[4]Key Laboratory of AIDS Immunology, Chinese Academy of Medical Sciences, Shenyang 110001, China

[5]Collaborative Innovation Center for Diagnosis and Treatment of Infectious Diseases, 79 Qingchun Street, Hangzhou 310003, China

***Correspondce:** gxliang@cmu.edu.cn

Supplementary Figures 1-9
Supplementary Tables 1-2

Supplementary Fig. 1

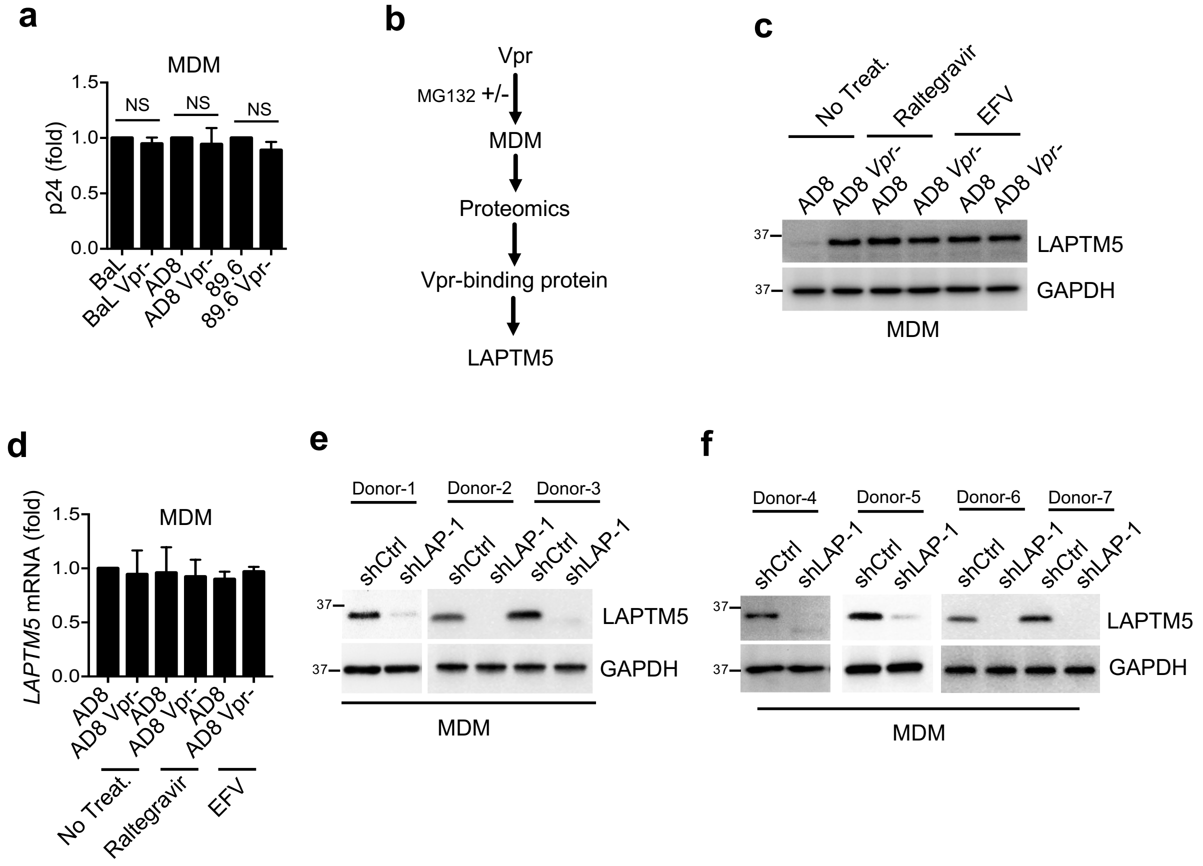

Supplementary Figure 1. LAPTM5 restricts HIV-1 infection in macrophages and is counteracted by Vpr.

a, MDMs were infected with 100 ng of replication-competent wild-type or Vpr-defective HIV-1 for 6 days. At 2 dpi, cells were treated with 2 μM raltegravir to block subsequent rounds of infection. Virion capsids in culture were measured by p24 ELISA. **b,** Schematic representation of the experimental design used to identify LAPTM5 in MDMs. **c,d,** MDMs infected with 100 ng of wild-type or Vpr-defective HIV-1_{AD8} were treated with or without raltegravir (2 μM) or EFV (0.3 μM) 6 h after infection. At 6 dpi, cells were lysed for western blotting to assess LAPTM5 and GAPDH expression (c). *LAPTM5* transcript levels were measured by qPCR (d), normalized by *GAPDH*. Data are plotted as mean ± SEM of three independent experiments. **e,f,** Western blot of Fig. 1a-c. All blotting data are representative of three independent experiments; their full-size images are presented in the Source Data.

Supplementary Fig. 2

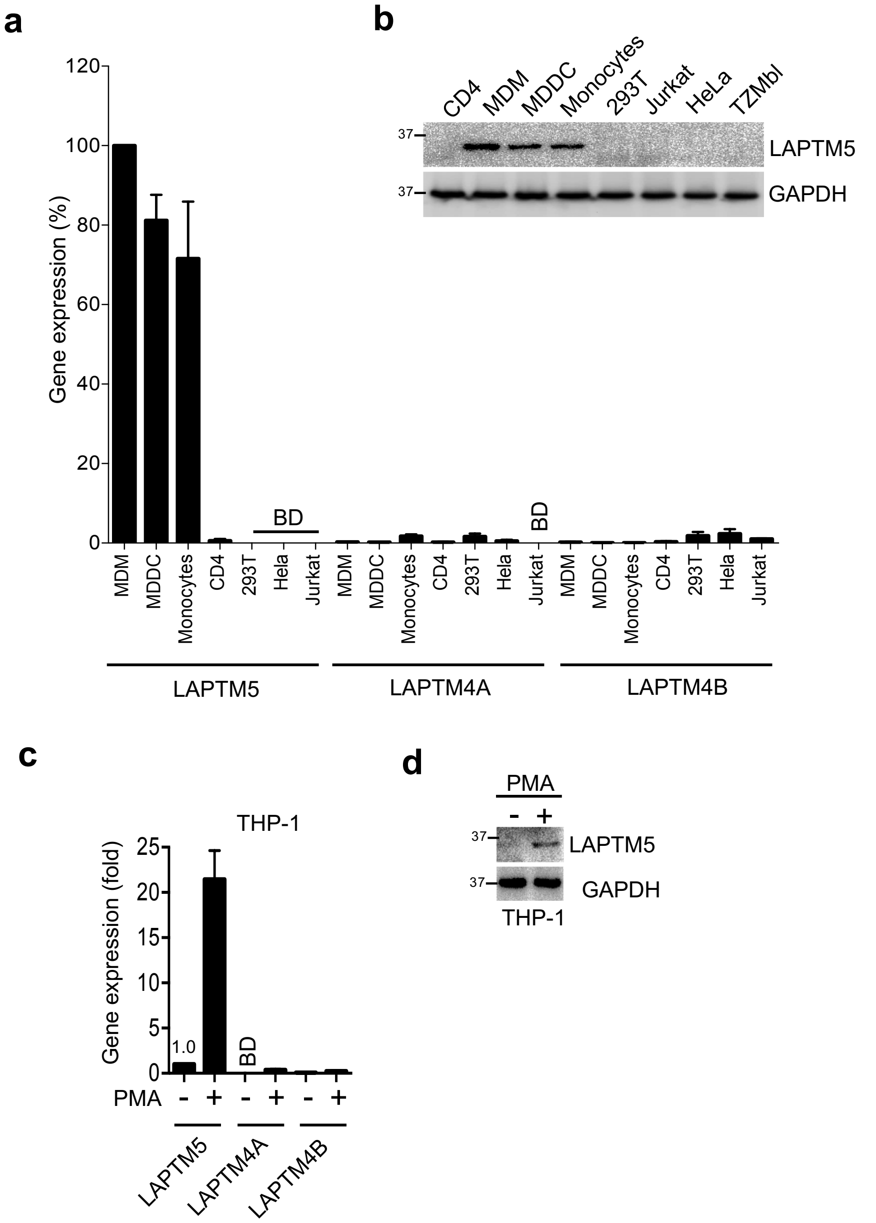

Supplementary Figure 2. LAPTM5 expression profiling in host cells.

a,b, Total RNA was extracted from primary monocytes, MDMs, MDDCs, stimulated CD4$^+$ T cells, established cell lines 293T cells, HeLa cells, and Jurkat cells. Quantitative PCR was used to measure *LAPTM5*, *4A*, and *4B* transcript levels normalized to *GAPDH* levels (a) and western blotting was conducted to assess LAPTM5 and GAPDH expression (b). **c,d,** Total RNA was extracted from THP-1 cells stimulated with or without PMA. Quantitative PCR was used to measure *LAPTM5*, *4A*, and *4B* transcript levels normalized to *GAPDH* levels (c), and western blotting was performed to assess LAPTM5 and GAPDH expression (d). BD, below the detection limit. Data are plotted as mean ± SEM of three independent experiments. All blotting data are representative of three independent experiments; their full-size images are presented in the Source Data.

Supplementary Fig. 3

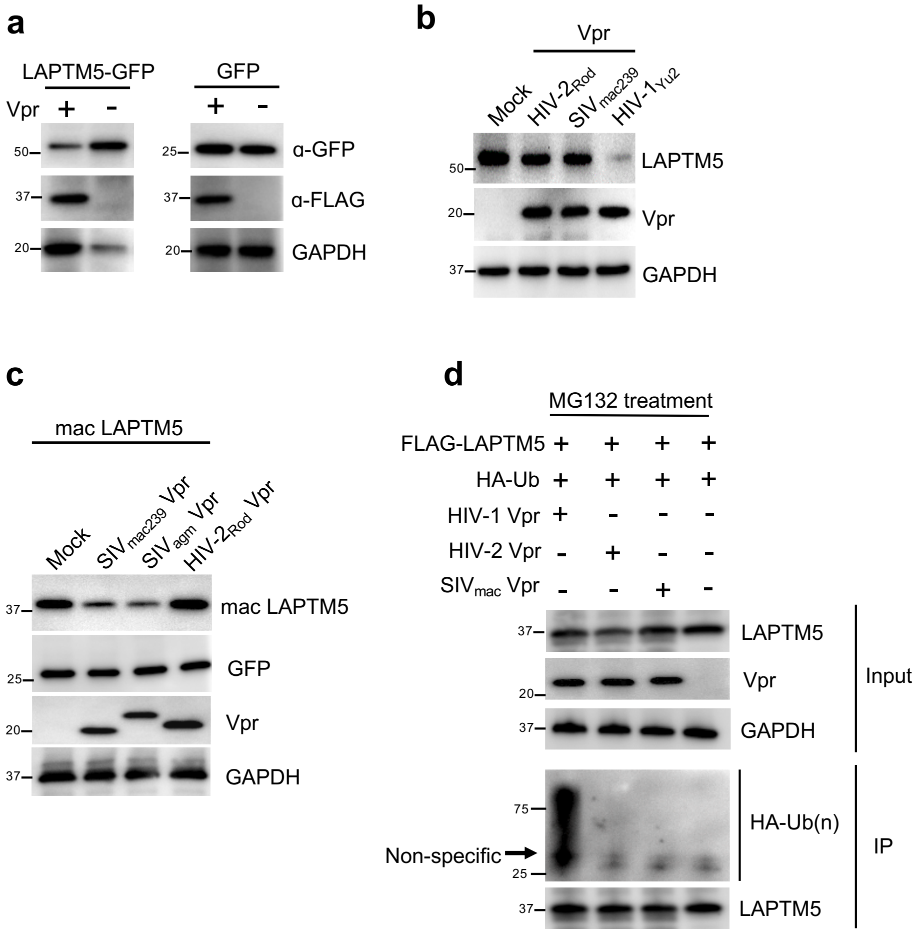

Supplementary Figure 3. Vpr derived from SIV and HIV-2 could not efficiently induce degradation of human LAPTM5 protein.

a, 293T cells were cotransfected with GFP-tagged LAPTM5 or mock (GFP) expression constructs with or without a FLAG-tagged Vpr of NL4-3 expression vector. Two days after transfection, cells were lysed for western blotting to assess LAPTM5, Vpr, and GAPDH expression. **b,** HeLa cells were cotransfected with a GFP-tagged LAPTM5 expression construct with or without FLAG-tagged Vpr expression vectors derived from HIV-2_{Rod}, SIV_{mac239}, or HIV-1_{Yu2}. Two days after transfection, cells were lysed for western blotting to assess LAPTM5, Vpr, and GAPDH expression. **c,** HeLa cells were cotransfected with the expression vectors of Vpr derived from SIV_{mac239}, SIV_{agm}, or HIV-2_{Rod} or mock, along with rhesus macaque (mac) LAPTM5 expression constructs in the presence of a GFP-monitoring expression vector. At 48 h after cotransfection, cells were lysed for western blotting to assess LAPTM5, Vpr, GFP, and GAPDH expression. **d,** 293T cells were cotransfected with or without a FLAG-tagged LAPTM5, HA-Tagged Ub, and GFP-Tagged Vpr from HIV-$1_{NL4\text{-}3}$, HIV-2_{Rod}, or SIV_{mac239} expression vectors, as indicated. At 24 h after cotransfection, cells were treated with MG132 (1.5 μM) for 10 h and then lysed for IP assays and treated with anti-FLAG antibody-conjugated M2 agarose beads to precipitate LAPTM5. Western blotting was conducted to detect ubiquitinated species as indicated, LAPTM5, Vpr, and GAPDH. All blotting data are representative of three independent experiments; their full-size images are presented in the Source Data.

Supplementary Fig. 4

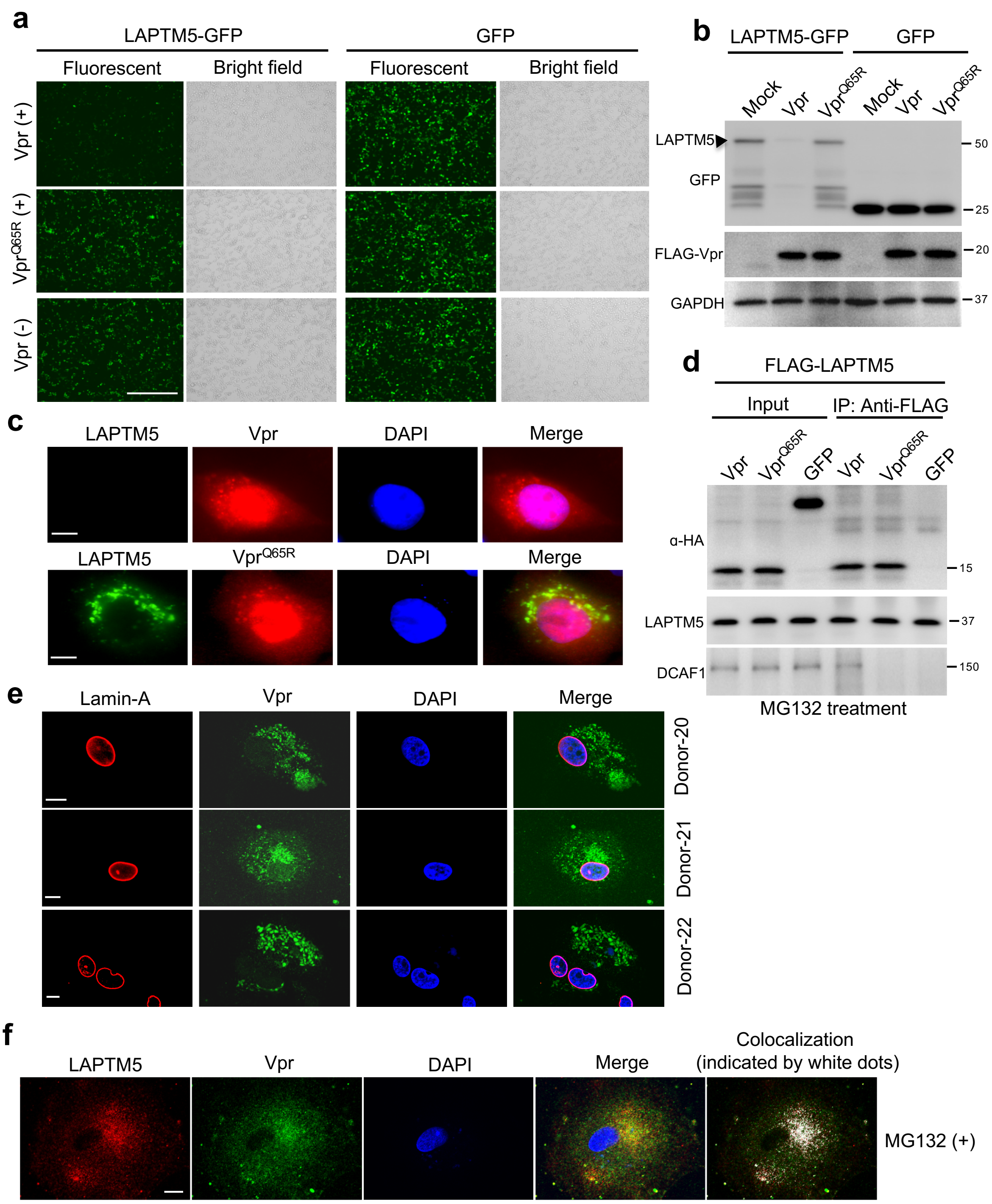

Supplementary Figure 4. Colocalization of Vpr with LAPTM5.

a, HeLa cells were cotransfected with GFP-tagged LAPTM5 or mock (GFP) expression constructs with or without FLAG-tagged wild-type Vpr or Vpr^{Q65R} mutant expression vectors derived from NL4-3. At 24 h after transfection, GFP positive cells were visualized. Scale bars, 400 μm. **b,** HeLa cells were cotransfected with GFP-tagged LAPTM5 or mock (GFP) expression constructs with or without FLAG-tagged Vpr or Vpr^{Q65R} mutant expression vectors. Two days after transfection, cells were lysed to assess LAPTM5-GFP, GFP, Vpr, and GAPDH expression by western blotting. **c,** HeLa cells were cotransfected with a GFP-tagged LAPTM5 expression construct along with FLAG-tagged wild-type Vpr or Vpr^{Q65R} mutant expression vectors derived from NL4-3. At 24 h after transfection, cells were fixed and immunostained with anti-FLAG antibodies, which were probed with a secondary antibody conjugated with Alexa Fluor 555, and the cell nuclei were stained with DAPI. Scale bars, 10 μm. **d,** HeLa cells were cotransfected with a FLAG-tagged LAPTM5 expression construct along with HA-tagged wild-type Vpr or Vpr^{Q65R} mutant or HA-Tagged GFP expression vectors. Two days after cotransfection, cells were treated with MG132 and lysed for IP assays with anti-FLAG antibody-conjugated M2 agarose beads to precipitate LAPTM5. Western blotting was performed to detect Vpr, LAPTM5, and DCAF1 using specific antibodies. **e,** MDMs were transduced with a lentiviral FLAG-tagged Vpr expression construct. After puromycin selection, cells were fixed and immunostained with anti-FLAG or anti-Lamin-A antibodies that were probed with a secondary antibody conjugated with Alexa Fluor 488 or 555, and the cell nuclei were stained with DAPI. Scale bars, 10 μm. **f,** MDMs were transduced with a lentiviral FLAG-tagged Vpr expression construct. After puromycin selection, cells were treated with MG132 for 12 h (at 1.0 μM), fixed, and immunostained with anti-FLAG or anti-LAPTM5 antibodies that were probed with a secondary antibody conjugated with Alexa Fluor 488 or 555, and the cell nuclei were stained with DAPI. The white dots represent the colocalized LAPTM5 and Vpr proteins which were visualized using the Image J software. Scale bars, 10 μm. Notably, primary MDMs non-specifically stained with the secondary antibodies were not observed. All data are representative of three independent experiments.

Supplementary Fig. 5

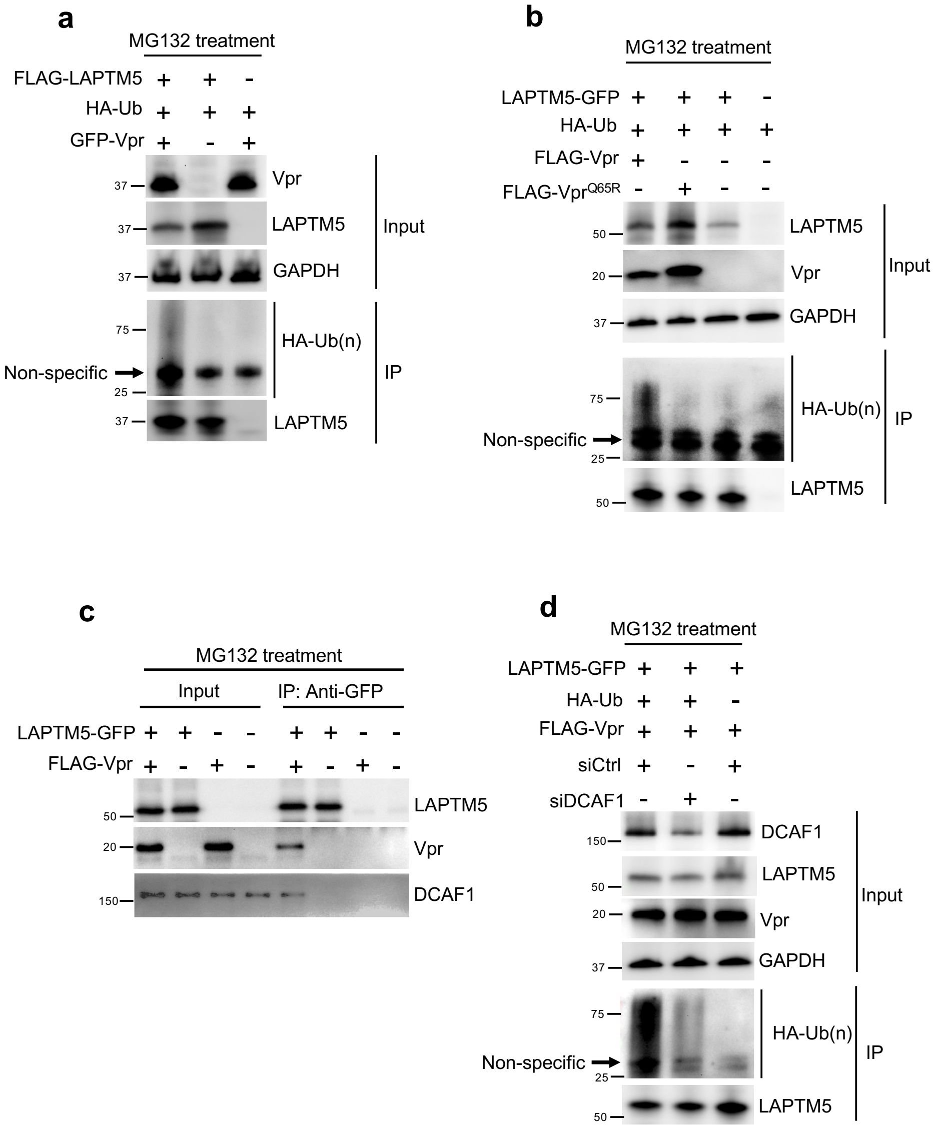

Supplementary Figure 5. Vpr promotes polyubiquitination of LAPTM5.
a, HeLa cells were cotransfected with or without FLAG-tagged LAPTM5, GFP-tagged wild-type Vpr, and HA-Tagged Ub expression vectors, as indicated. At 24 h after cotransfection, cells were treated with MG132 (at 1.5 μM) for 10 h and, subsequently, lysed for IP assays with anti-FLAG antibody-conjugated M2 agarose beads to precipitate LAPTM5. Western blotting was conducted to detect ubiquitinated species as indicated, LAPTM5, Vpr, and GAPDH with specific antibodies. **b,** HeLa cells were cotransfected with or without GFP-tagged LAPTM5, HA-Tagged Ub, and FLAG-Tagged Vpr or its mutant Vpr^{Q65R} expression construct, as indicated. At 24 h after cotransfection, cells were treated with MG132 (at 1.5 μM) for 10 h and subsequently lysed for IP assays with anti-GFP antibodies to precipitate LAPTM5. Western blotting was conducted to detect the ubiquitinated species as indicated, LAPTM5, DCAF1, Vpr, and GAPDH with their specific antibodies. **c,** HeLa cells were cotransfected with or without a GFP-tagged LAPTM5 expression construct in the presence or absence of a FLAG-tagged Vpr expression vector. At 24 h after cotransfection, cells were treated with MG132 (at 1.5 μM) for 10 h and subsequently lysed for IP assays with anti-GFP antibodies. Western blotting was conducted to detect LAPTM5, Vpr, and DCAF1 by their specific antibodies. **d,** HeLa cells were cotransfected with or without GFP-tagged LAPTM5, FLAG-tagged wild-type Vpr, and HA-Tagged Ub expression constructs in the presence of absence of siRNA against DCAF1 or control siRNA, as indicated. At 24 h after cotransfection, cells were treated with MG132 (1.5 μM) for 10 h and subsequently lysed for IP assays with anti-GFP antibodies to precipitate LAPTM5. Western blotting was conducted to detect ubiquitinated species as indicated, LAPTM5, DCAF1, Vpr, and GAPDH with specific antibodies. All blotting data are representative of three independent experiments; their full-size images are presented in the Source Data.

Supplementary Fig. 6

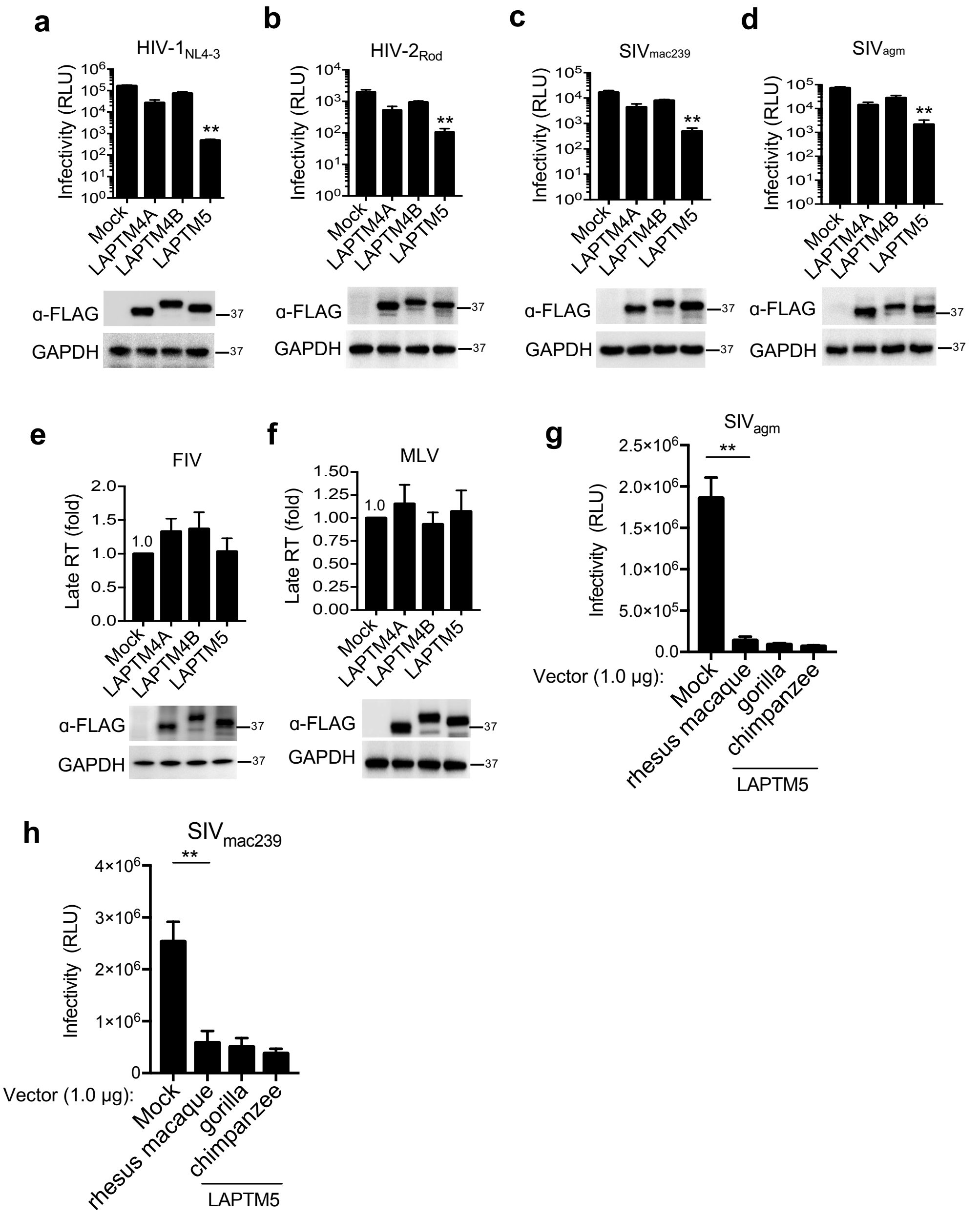

Supplementary Figure 6. LAPTM5 restricts HIV-1, HIV-2, and SIV infection.
a–**f,** HeLa cells were cotransfected with FLAG-tagged LAPTM5, 4A, 4B, or mock expression constructs along with HIV-1_{NL4-3} (**a**), HIV-2_{Rod} (**b**), SIV_{mac239} (**c**), SIV_{agm} (**d**), FIV-14 (**e**), or MLV (**f**) proviral vectors. Two days after transfection, TZM-bl reporter cells were used to measure viral infectivity. MOLT-4 cells were infected with FIV-14, or mouse NIH3T3 cells were infected with MLV, and the late reverse-transcript products were measured by qPCR. Western blotting was performed to assess LAPTM5, 4A, 4B, and GAPDH expression. **P <0.01 (two-tailed, unpaired Student's t-test), data are plotted as mean ± SEM of three independent experiments. **g,h,** HeLa cells were cotransfected with different species of LAPTM5 expression vectors and replication-competent proviral vectors of SIV as indicated. At 48 h after transfection, the produced virion infectivity was measured using TZM-bl reporter cells. **P <0.01 (two-tailed, unpaired Student's t-test), data are plotted as mean ± SEM of three independent experiments. All western blot data are representative of three independent experiments; their full-size images are presented in the Source Data.

Supplementary Fig. 7

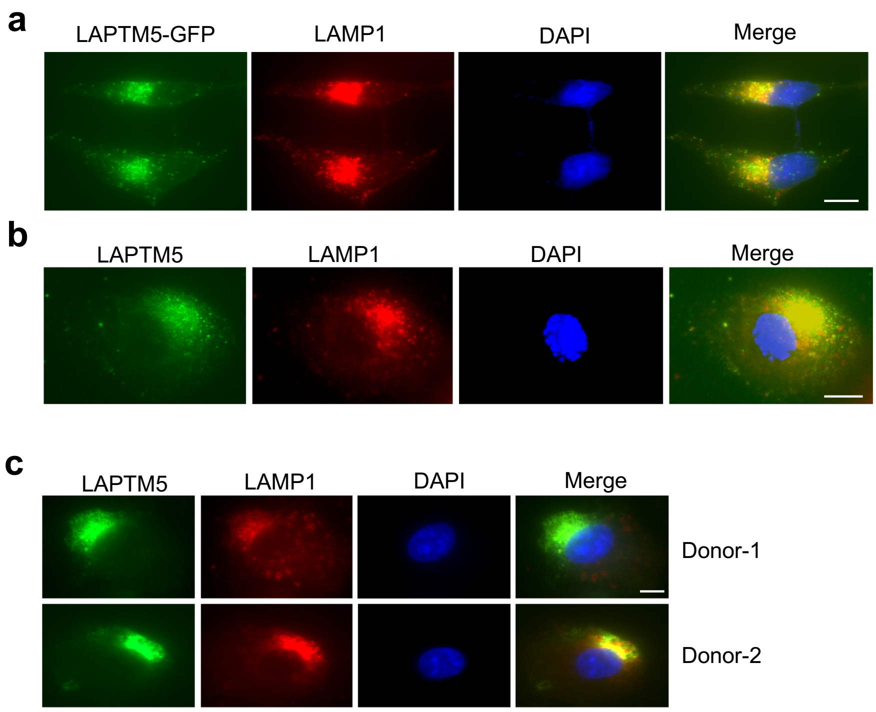

Supplementary Figure 7. IF-based analyses of subcellular localization of LAPTM5.
a, HeLa cells that exogenously expressed LAPTM5-GFP were fixed and immunostained with an anti-LAMP1 antibody. **b,** PMA-stimulated THP-1 cells were fixed and immunostained with anti-LAPTM5 or anti-LAMP1 antibodies. **c,** Primary MDMs were fixed and immunostained with anti-LAPTM5 or anti-LAMP1 antibodies. All primary antibodies were probed with a secondary antibody conjugated with Alexa Fluor 488 or 555. Cell nuclei were stained with DAPI. Scale bars, 10 μm. All data are representative of three independent experiments.

Supplementary Fig. 8

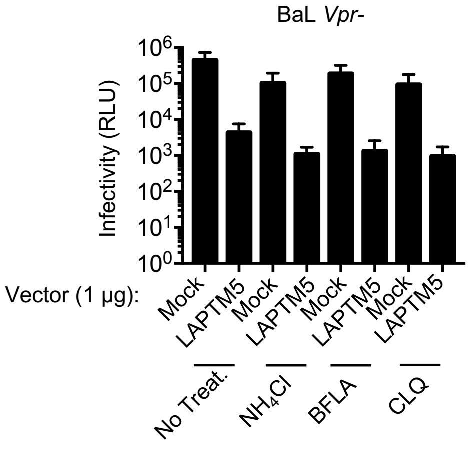

Supplementary Figure 8. Lysosomal inhibitors do not abolish the inhibitory effect of LAPTM5 on HIV-1 infectivity.

HeLa cells were cotransfected with FLAG-tagged LAPTM5 or mock expression constructs along with Vpr-defective HIV-1_{BaL} in the absence or presence of lysosomal inhibitors (treatment for the last 24 h). Two days after transfection, TZM-bl reporter cells were used to measure viral infectivity. Data are plotted as mean ± SEM of three independent experiments.

Supplementary Fig. 9

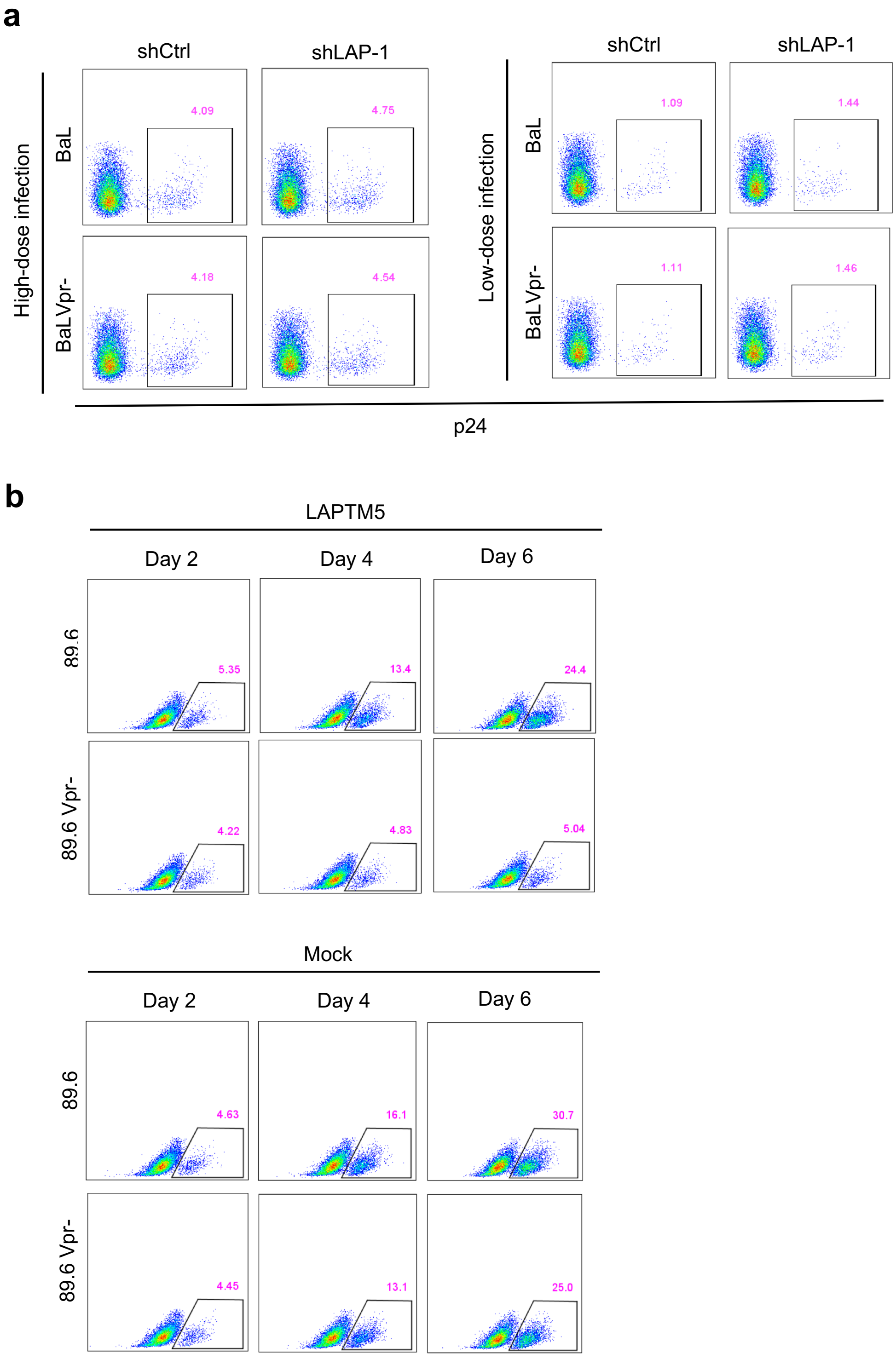

Supplementary Figure 9. Infected population of macrophages and CD4$^+$ T cells.
a, MDM cells were infected with wild-type or Vpr-defective HIV-1$_{BaL}$ under the same experiment condition as in Fig. 6a–c. Six days after transfection, p24 positive cells were measured. **b,** CD4$^+$ T cells were infected with wild-type or Vpr-defective HIV-1$_{89.6}$ under the same experiment condition as in Figs.7b–c. Two, four, or six days after transfection, p24 positive cells were measured. All FACS data are representative of three independent experiments.

Supplementary Table 1

Exp-1 (from 5 donors)

Accession (MG132+)	Name	Peptides amount	Accession (MG132-)	Name	Peptides amount
Q15149	PLEC	241	Q15149	PLEC	242
P35579	MYH9	172	P35579	MYH9	180
P21333	FLNA	93	P21333	FLNA	100
P46940	IQGAP1	87	P46940	IQGAP1	92
P08670	Vimentin	85	P08670	Vimentin	79
Q92614	MYO18A	77	P12814	ACTN1	78
P12814	ACTN1	72	Q00610	CLTC	78
Q00610	CLTC	71	O43707	ACTN4	73
O43707	ACTN4	69	Q92614	MYO18A	71
Q9Y4I1	MYO5A	67	P60709	ACTB	61
O00160	MYO1F	62	O00160	CLTC	61
P60709	ACTB	55	Q9Y4I1	MYO5A	61
Q12965	MYO1E	54	O00159	MYO1C	56
O00159	MYO1C	51	Q12965	MYO1E	55
B0I1T2	MYO1G	45	B0I1T2	MYO1G	53
P06396	GSN	43	Q9UHB6	LIMA1	46
P68032	ACTC1	41	P06396	GSN	44
O94832	MYO1D	41	P68032	ACTC1	42
Q9UHB6	LIMA1	40	Q13045	LIMA1	42
Q13571	LAPTM5	40	O75369	FLNB	42
Q8NF50	DOCK8	38	Q8NF50	DOCK8	41
P63010	AP2B1	37	P63010	AP2B1	40
Q10567	AP1B1	35	O95425	SVIL	40
O95782	AP2A1	34	Q13813	SPTAN1	37
O43795	MYO1B	34	O94832	MYO1D	36
P13796	LCP1	33	Q10567	AP1B1	35
P83111	LACTB	33	Q9UM54	MYO6	34
P04264	KRT1	31	P13796	LCP1	33
O95425	SVIL	31	O95782	AP2A1	32
O75369	FLNB	31	P83111	LACTB	30
P40939	HADHA	30	O43795	MYO1B	30
Q9UM54	MYO6	30			

Exp-2 (from 5 donors)

Accession (MG132+)	Name	Peptides amount	Accession (MG132-)	Name	Peptides amount
Q15149	PLEC	190	Q15149	PLEC	185
P35579	MYH9	109	P35579	MYH9	123
Q00610	CLTC	103	Q00610	CLTC	109
P08670	Vimentin	77	P08670	Vimentin	82
P46940	IQGAP1	73	P46940	IQGAP1	67
P21333	FLNA	61	P21333	FLNA	66
O00159	MYO1C	48	Q92614	MYO18A	54
P12814	ACTN1	47	O43707	ACTN4	53
Q9Y4I1	MYO5A	46	P12814	ACTN1	52
O43707	ACTN4	45	Q12965	MYO1E	50
Q13571	LAPTM5	44	Q9Y4I1	MYO5A	48
Q92614	MYO18A	41	O00159	MYO1C	44
Q12965	MYO1E	37	O94832	MYO1D	43
O94832	MYO1D	37	Q8NF50	DOCK8	43
P60709	ACTB	34	P60709	ACTB	41
B0I1T2	MYO1G	33	P68133	ACTA1	35
P02545	LMNA	31	B0I1T2	MYO1G	35
			P02545	LMNA	35
			P63267	ACTG2	34
			Q96N67	DOCK7	32
			P63010	AP2B1	31

Supplementary Table 1. Candidate list of Vpr-interacting partners in MDMs from two independent experiments.

Supplementary Table 2. **Primer sequences in this study**

Name		Sequence (5'→3')
FIV for		5′-GTATGATCGTACTCATCCTCCTGAT
FIV rev		5′-TCTACATTGCATTCTGGCTGGT
MLV for		5′-CGTCAGCGGGGGTCTTTC
MLV rev		5′-CTGGGCAGGGGTCTCCCG
GAPDH for		5′-AATGACCCCTTCATTGAC
GAPDH rev		5′-TCCACGACGTACTCAGCGC
LAPTM5 for		5′-GCGTCTTGTTGTTCATCGAGC
LAPTM5 rev		5′-CGATCCTGAGGTAGCCCAT
LAPTM4A for		5′-ATGGTGTCCATGAGTTTCAAGC
LAPTM4A rev		5′-CCACAGTCAGCAAAATTGCCA
LAPTM4B for		5′-GCCCGGAGCGATGAAGATG
LAPTM4B rev		5′-CAACAGTACCACAGCATTGATGA
