# Supplementary material for: Vpr counteracts the restriction of LAPTM5 to promote HIV-1 infection in macrophages
Source: Nat Commun. 2021 Jun 17;12:3691. doi: 10.1038/s41467-021-24087-8 (PMC8211709; doi:10.1038/s41467-021-24087-8)
Supplement: Supplementary file 3 — Reporting Summary [file 41467_2021_24087_MOESM3_ESM.pdf]

nature research

Corresponding author(s): Guoxin Liang

Last updated by author(s): 2021/05/18

Reporting Summary

Nature Research wishes to improve the reproducibility of the work that we publish. This form provides structure for consistency and transparency in reporting. For further information on Nature Research policies, see Authors & Referees and the Editorial Policy Checklist .

Statistics

For all statistical analyses, confirm that the following items are present in the figure legend, table legend, main text, or Methods section.

n/a	Confirmed	
☐	☒	The exact sample size (n) for each experimental group/condition, given as a discrete number and unit of measurement
☐	☒	A statement on whether measurements were taken from distinct samples or whether the same sample was measured repeatedly
☐	☒	The statistical test(s) used AND whether they are one- or two-sided<br>*Only common tests should be described solely by name; describe more complex techniques in the Methods section.*
☒	☐	A description of all covariates tested
☒	☐	A description of any assumptions or corrections, such as tests of normality and adjustment for multiple comparisons
☐	☒	A full description of the statistical parameters including central tendency (e.g. means) or other basic estimates (e.g. regression coefficient) AND variation (e.g. standard deviation) or associated estimates of uncertainty (e.g. confidence intervals)
☐	☒	For null hypothesis testing, the test statistic (e.g. F, t, r) with confidence intervals, effect sizes, degrees of freedom and P value noted<br>*Give P values as exact values whenever suitable.*
☒	☐	For Bayesian analysis, information on the choice of priors and Markov chain Monte Carlo settings
☒	☐	For hierarchical and complex designs, identification of the appropriate level for tests and full reporting of outcomes
☒	☐	Estimates of effect sizes (e.g. Cohen's d, Pearson's r), indicating how they were calculated

Our web collection on statistics for biologists contains articles on many of the points above.

Software and code

Policy information about availability of computer code

Data collection	We do not have used specific softwares to collect data.
Data analysis	Data were analyzed with GraphPad Prism 6.0 Software for statistics. Image J software was used to analyze the colocalization between LAPTM5 and Vpr for micrographs in Supplementary Figure 4f.

For manuscripts utilizing custom algorithms or software that are central to the research but not yet described in published literature, software must be made available to editors/reviewers. We strongly encourage code deposition in a community repository (e.g. GitHub). See the Nature Research guidelines for submitting code & software for further information.

Data

Policy information about availability of data

All manuscripts must include a data availability statement. This statement should provide the following information, where applicable:

- Accession codes, unique identifiers, or web links for publicly available datasets
- A list of figures that have associated raw data
- A description of any restrictions on data availability

Data supporting the findings of this study are available within the article and its Supplementary information files, and from the corresponding authors upon reasonable request. Liquid chromatography and mass spectrometry data are available in Mendeley Data (https://data.mendeley.com/datasets/62tgy3jbtg/1). Source Data are provided with this paper.

Field-specific reporting

Please select the one below that is the best fit for your research. If you are not sure, read the appropriate sections before making your selection.

Life sciences study design

All studies must disclose on these points even when the disclosure is negative.

Sample size	The sample sizes were chosen on the basis of previous experience in accordance to the standards in the field. Experiments were performed at least 3 times to confirm reproducibility. Sample size information was described in Methods and Figure legends. which is also statistically significant using GraphPad Prism 6.0 software (two-tailed, unpaired Student's t-test), described in Figure legends sections and Methods.
Data exclusions	No data were excluded.
Replication	The data derived from cell lines were obtained from three independent experiment (SEM). The data derived primary cells isolated from healthy donors were generated from multiple independent experiments with independent donors (sample size, n was clearly described in Figure legends as well as Methods). All experiments were reproduced to reliably support conclusions stated in the manuscript and assay reproducibility is described in Figure legends.
Randomization	Samples were all randomly allocated to different experimental groups in this study. No specific randomization protocol has been used.
Blinding	Not applicable as the extracted results are objective in this study.

Reporting for specific materials, systems and methods

We require information from authors about some types of materials, experimental systems and methods used in many studies. Here, indicate whether each material, system or method listed is relevant to your study. If you are not sure if a list item applies to your research, read the appropriate section before selecting a response.

Materials & experimental systems

n/a	Involved in the study	
☐	☒	Antibodies
☐	☒	Eukaryotic cell lines
☒	☐	Palaeontology
☒	☐	Animals and other organisms
☐	☒	Human research participants
☒	☐	Clinical data

Methods

n/a	Involved in the study	
☒	☐	ChIP-seq
☒	☐	Flow cytometry
☒	☐	MRI-based neuroimaging

Antibodies

Antibodies used	The antibodies used in this study were as follows: polyclonal rabbit anti-LAPTM5 (Biorbyt, Cat. orb184851), monoclonal mouse anti-LAMP1 (Abcam, Cat.ab25630), polyclonal goat anti-gp120 (NIH AIDS Reagent Program), human monoclonal anti-gp41 (NIH AIDS Reagent Program), rabbit anti-GAPDH (Thermo, Cat.PA1-987), mouse monoclonal anti-FLAG (SIGMA, Cat.F1804), rabbit polyclonal anti-p24 (Abcam, Cat.ab63913), mouse monoclonal anti-GFP (Abmart, Cat.M20004), mouse monoclonal anti-HA (Abmart, Cat.M20003), mouse Igs-HRP (Abcam, Cat.6789), Higly corss-Adsorbed Alexa Fluor 488-, 647-, or 555-labeled goat anti-rabbit secondary antibody (Thermo, Cat.A32731; Cat.A32733; Cat.A32732), Highly corss-Adsorbed Alexa Fluor 488-, 647-, or 555-labeled goat anti-mouse secondary antibody (Thermo, Cat.A32723; CatA32728; CatA32727), rabbit and mouse IgG Trueblot (eBioscience, Cat.18-8816-33; Cat.18-8817-33), and rabbit or mouse IgG isotype control (Abcam, Cat. ab171870; Cat.18413). All of antibodies used in this study are also described in Methods in this manuscript.
Validation	All antibodies used in this study are commercial and well-established in the field. Validation data are provided for each antibody on the manufacturers' websites with antibody profiles and citations: polyclonal rabbit anti-LAPTM5 (Biorbyt, Cat. orb184851,1:1000), monoclonal mouse anti-LAMP1 (Abcam, Cat.ab25630, 1:1000, PMID: 32020711), polyclonal goat anti-gp120 (NIH AIDS Reagent Program, Cat.ARP-288, 1:20000, PMID: 25464830), human monoclonal anti-gp41 (NIH AIDS Reagent Program, Cat.ARP-11557, 1:5000, PMID: 25464830), rabbit anti-GAPDH (Thermo, Cat.PA1-987, 1:1000, PMID: 29726306), mouse monoclonal anti-FLAG (SIGMA, Cat.F1804, 1:1000, PMID: 25697406), rabbit polyclonal anti-p24 (Abcam, Cat.ab63913, 1:1000, PMID: 30081466), mouse monoclonal anti-GFP (Abmart, Cat.M20004, 1:2000), mouse monoclonal anti-HA (Abmart, Cat.M20003, 1:2000), mouse Igs-HRP (Abcam, Cat.6789, 1:5000, PMID: 32049000), Higly corss-Adsorbed Alexa Fluor 488-, 647-, or 555-labeled goat anti-rabbit secondary antibody (Thermo, Cat.A32731, 1:200, PMID: 30199819; Cat.A32733; 1:200, PMID: 29233834; Cat.A32732, 1:200, PMID: 28123570), Highly corss-Adsorbed Alexa Fluor 488-, 647-, or 555-labeled goat anti-mouse secondary antibody (Thermo, Cat.A32723, 1:200, PMID: 30214519; Cat.A32728; 1:200, PMID: 29490706; Cat.A32727, 1:200, PMID: 29653597), rabbit and mouse IgG Trueblot (eBioscience, Cat.18-8816-33,1:1000, PMID: 31941780; Cat.18-8817-33, 1:1000, PMID: 33411764), and rabbit or mouse IgG isotype control (Abcam, Cat. ab171870, PMID: 32206710;Cat.ab18413, PMID: 32157956).

Eukaryotic cell lines

Policy information about cell lines

Cell line source(s)	HEK293T, HeLa, mouse NIH3T3, MOLT4, Jurkat cell lines were obtained from American Type Culture Collection (ATCC, Manassas, VA, USA), and please see their catalog numbers in Methods. TZMbl cells were gifted by Dr.Guangxia Gao (PMID:30682371).
Authentication	Each cell line used was authenticated by Beijing Genetic Testing Biotechnology company using STR profiling method.
Mycoplasma contamination	We tested all of cell lines and no cell line found with any mycoplasma contamination.
Commonly misidentified lines (See ICLAC register)	No commonly misidentified cell lines were used.

Human research participants

Policy information about studies involving human research participants

Population characteristics	Healthy donors were randomly recruited and their blood samples were obtained to purify monocytes, CD4+T cell for this study. Please also see in Method section.
Recruitment	Healthy donors were randomly recruited through AIDS research center in The First Affiliated Hospital of China Medical University.
Ethics oversight	The Research and Ethics Commitee of The First Affiliated Hospital of China Medical University.

Note that full information on the approval of the study protocol must also be provided in the manuscript.
