# Supplementary material for: Vpr counteracts the restriction of LAPTM5 to promote HIV-1 infection in macrophages
Source: Nat Commun. 2021 Jun 17;12:3691. doi: 10.1038/s41467-021-24087-8 (PMC8211709; doi:10.1038/s41467-021-24087-8)

Supplementary uncropped blots

Fig. 1e

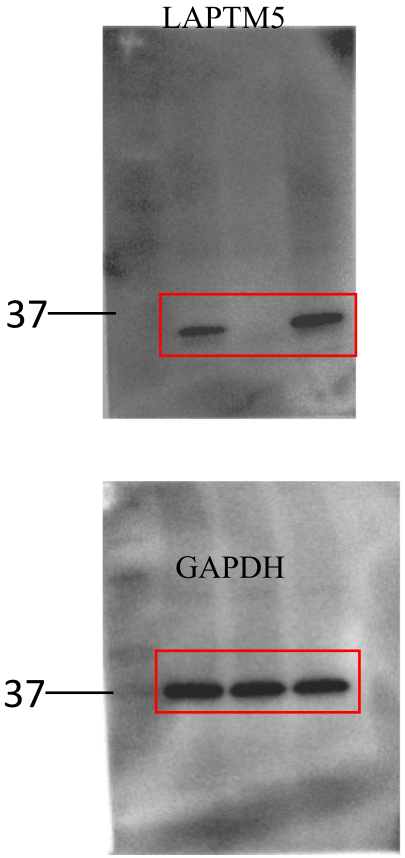

Fig. 2a

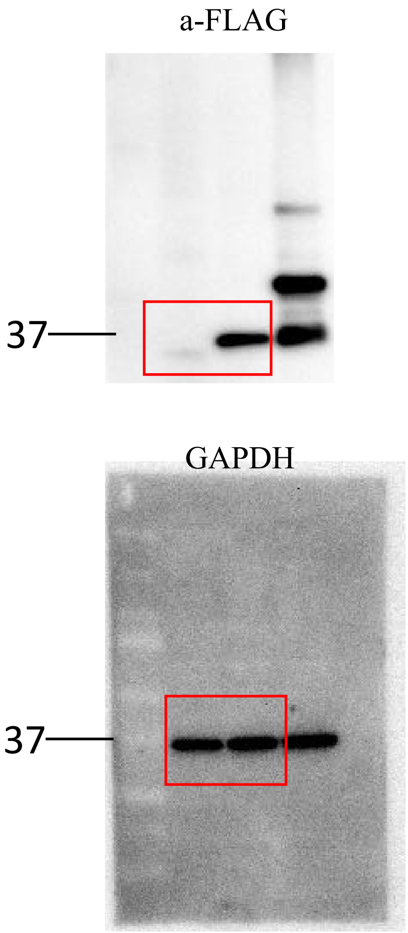

Fig. 2d

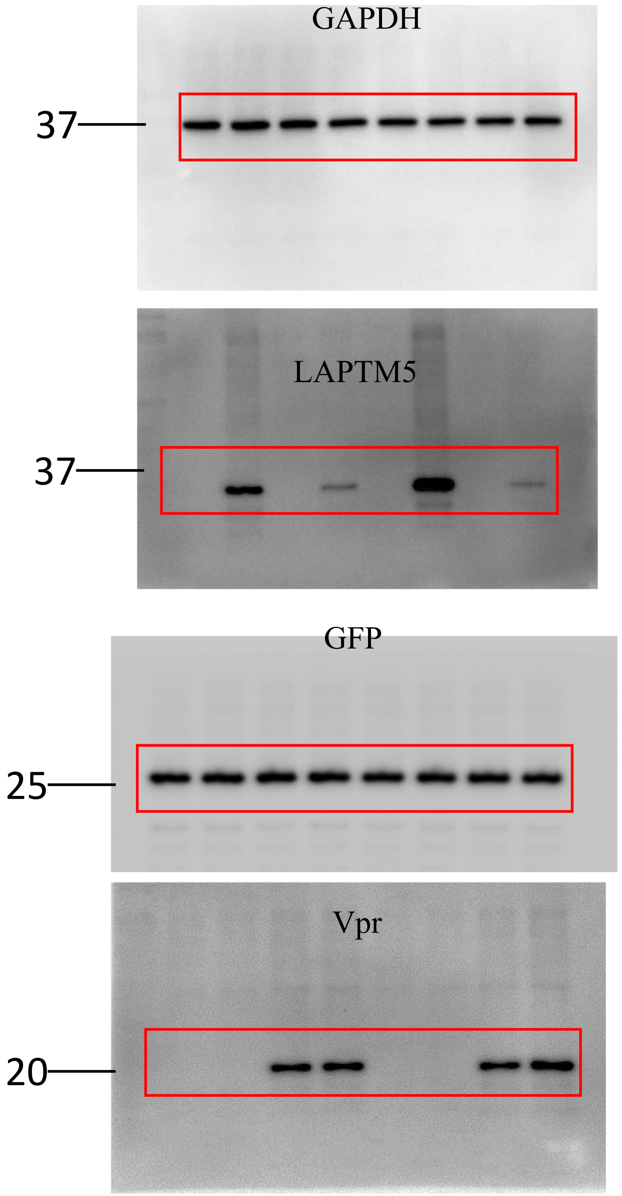

Fig. 3b

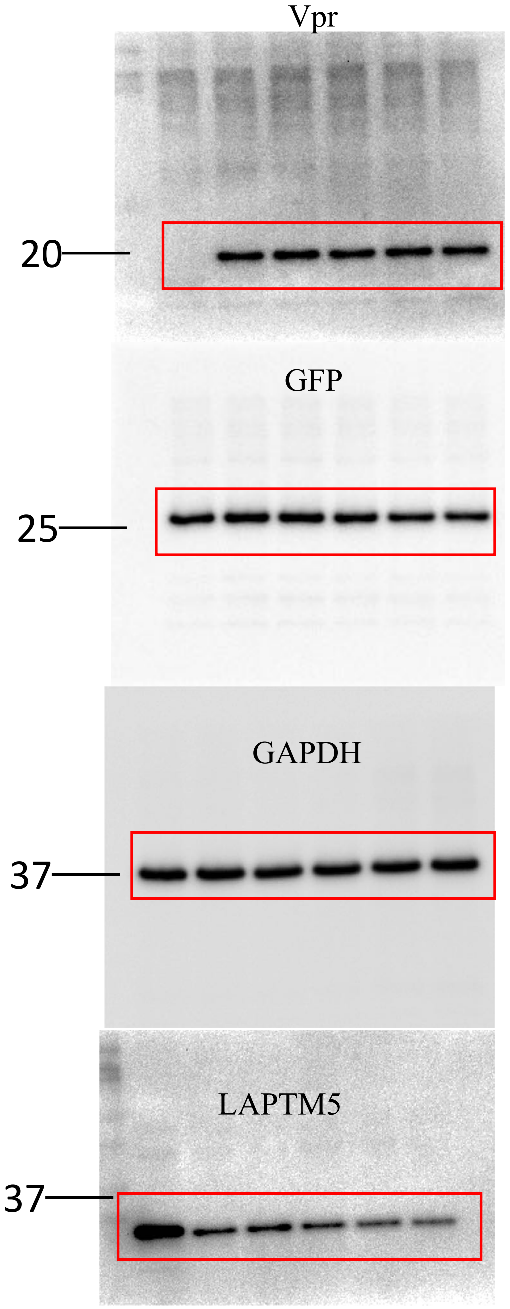

Fig. 3c

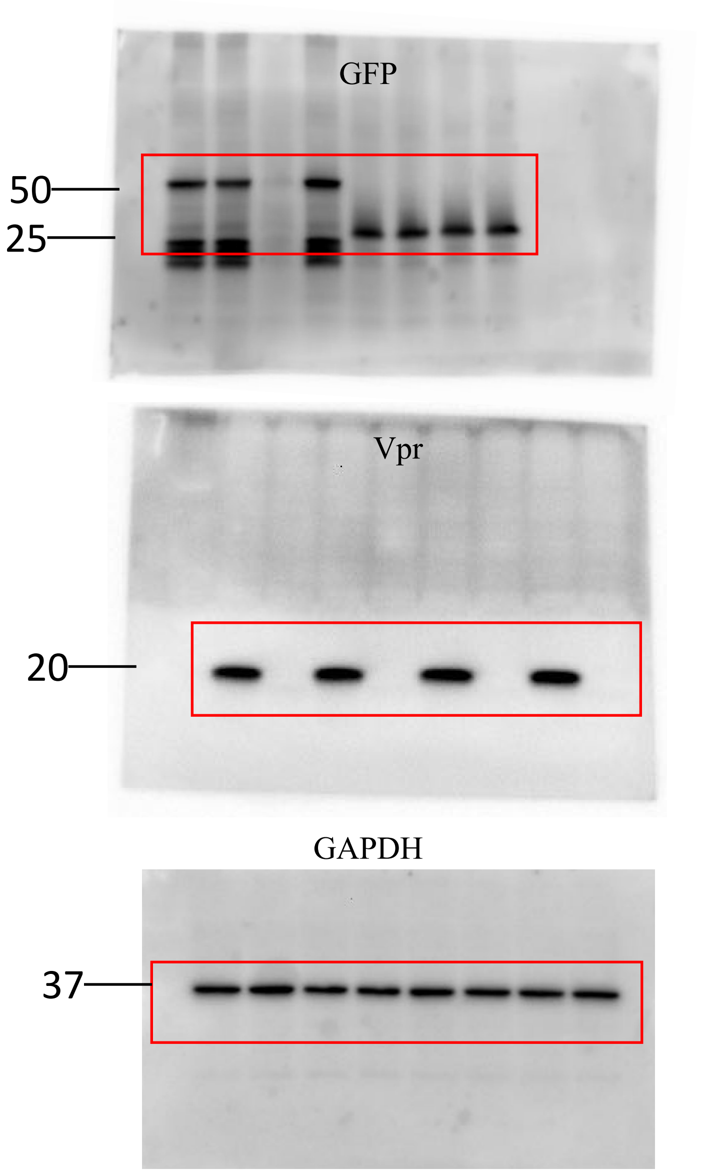

Fig. 3d

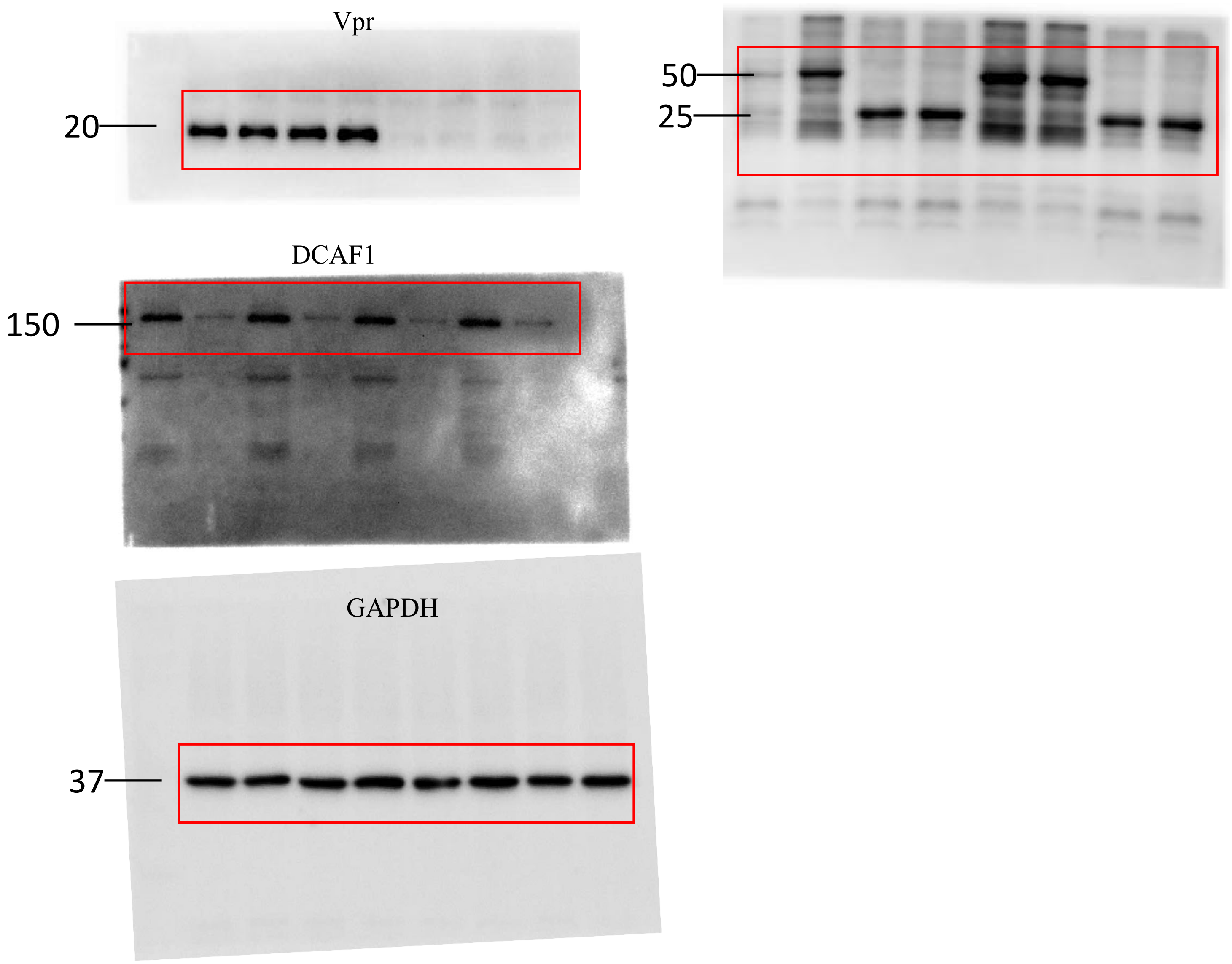
Vpr
20
DCAF1
150
GAPDH
37
GFP
50
25

Fig. 3e

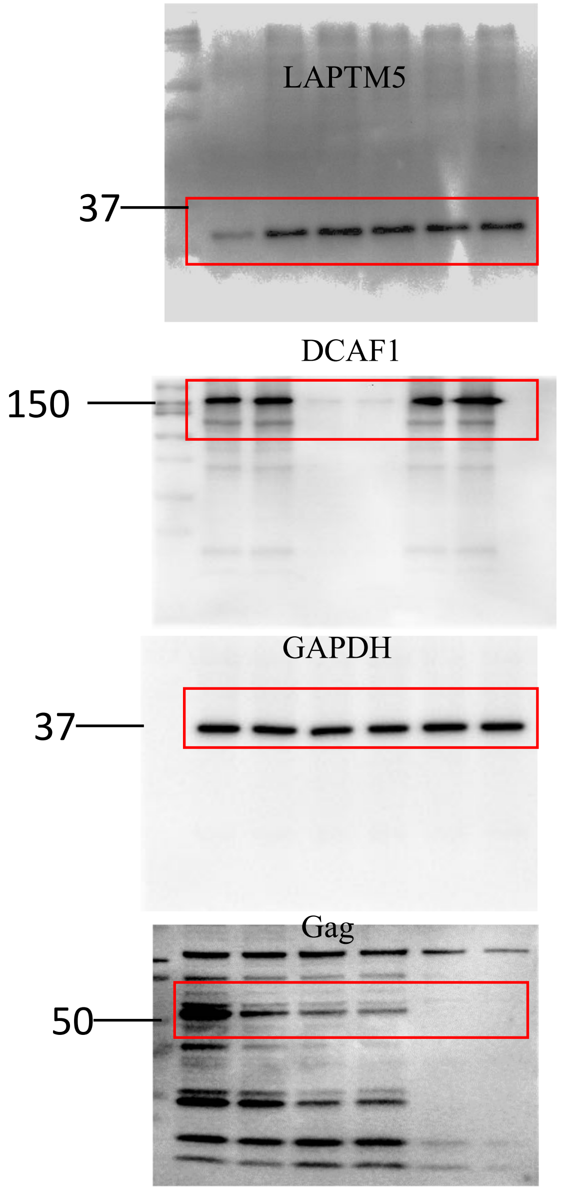
LAPTM5
37
DCAF1
150
GAPDH
37
Gag
50

Fig. 4a

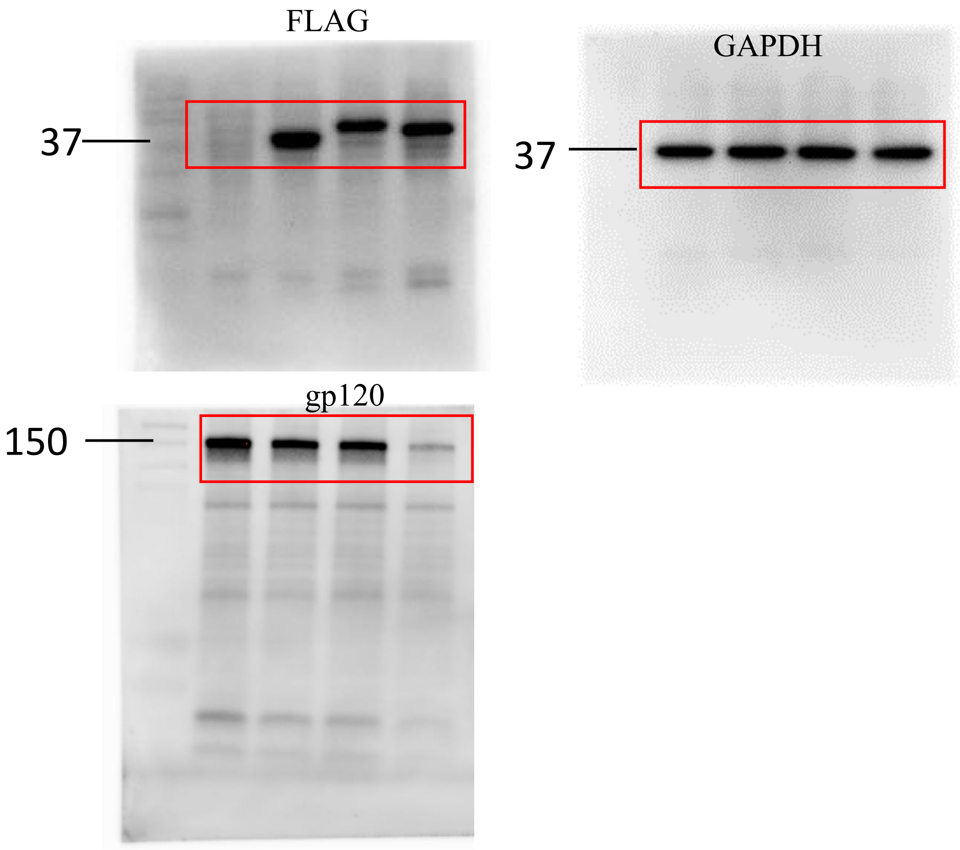

Fig. 4b

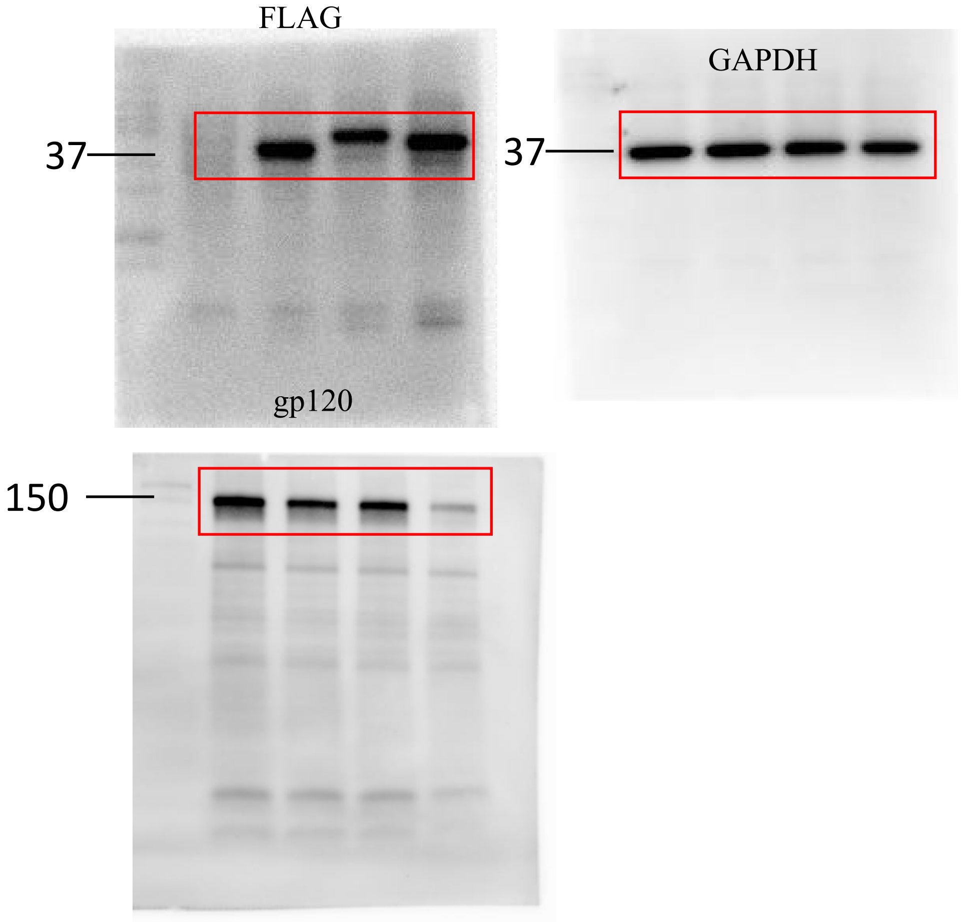

Fig. 4c

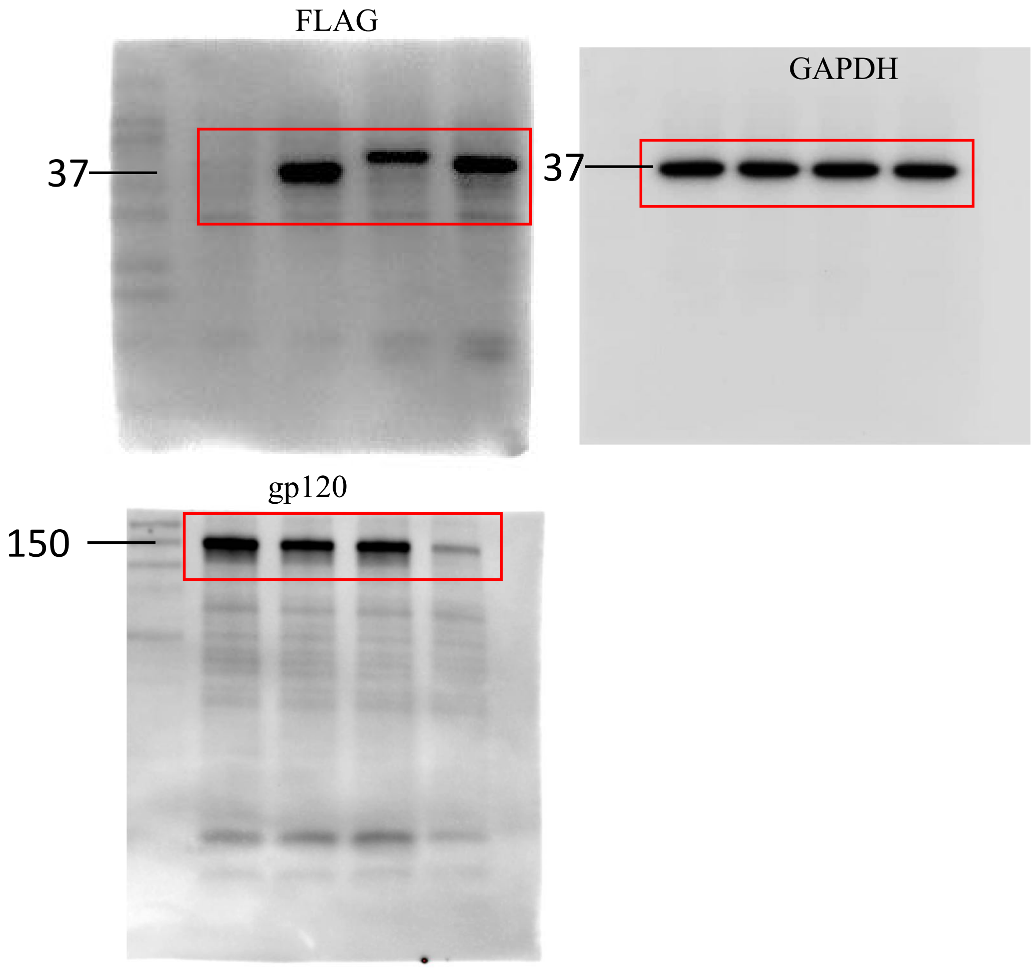

Fig. 5b, Left

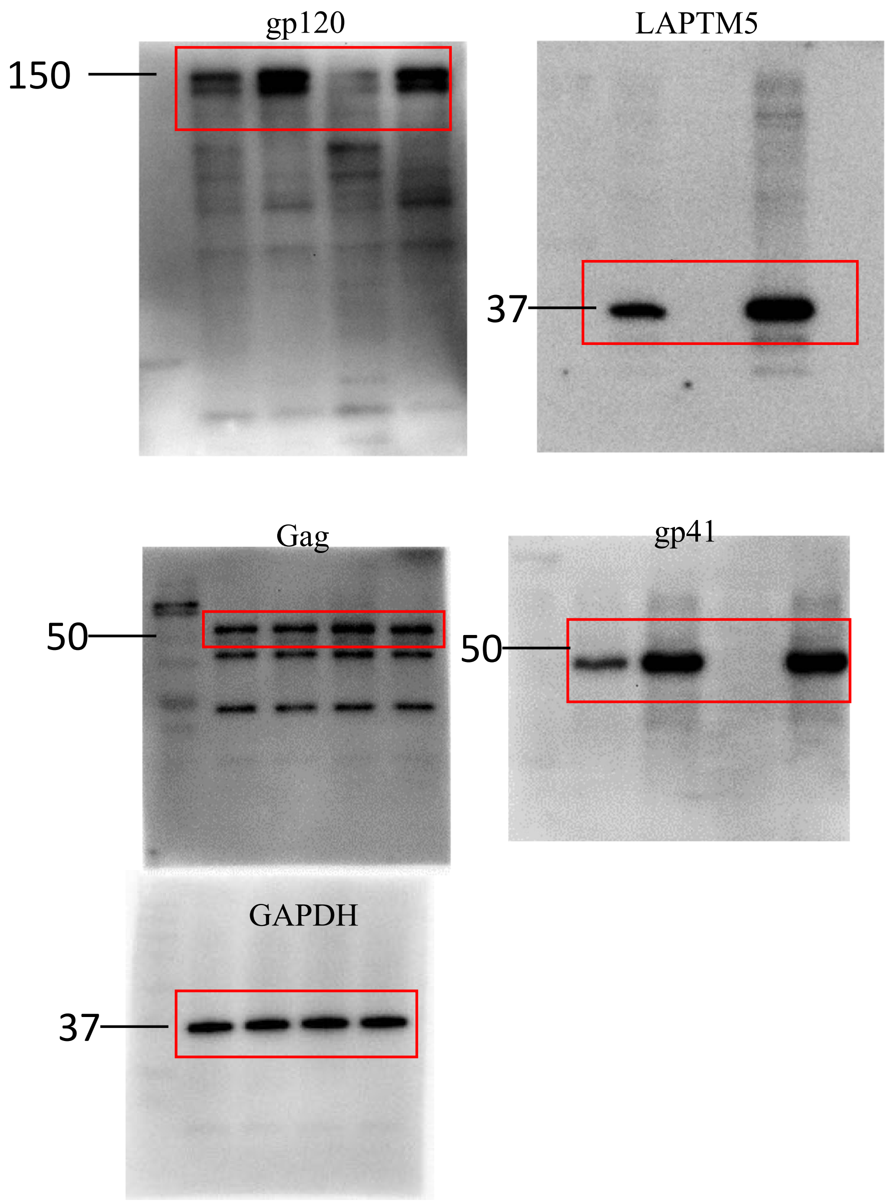

Fig. 5b, Right

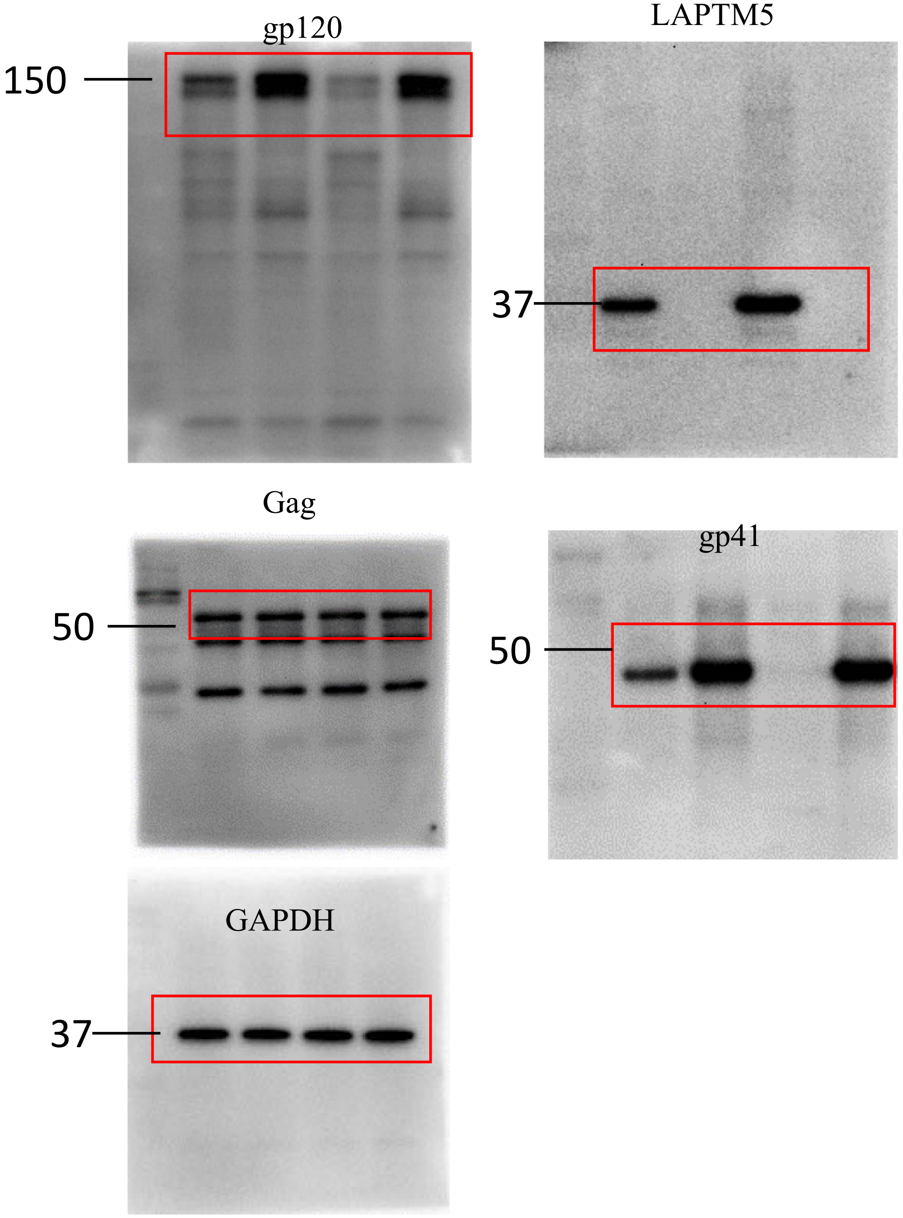

Fig. 5c

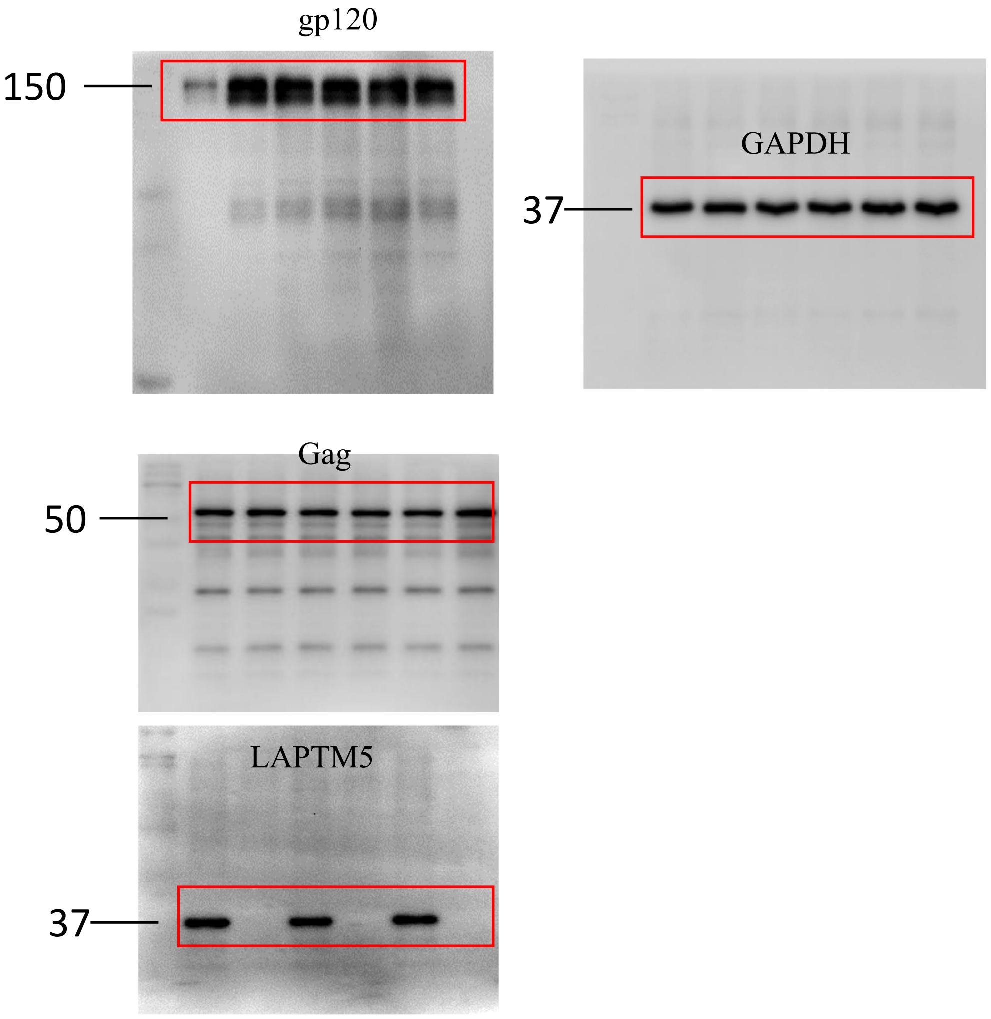

Fig. 5g

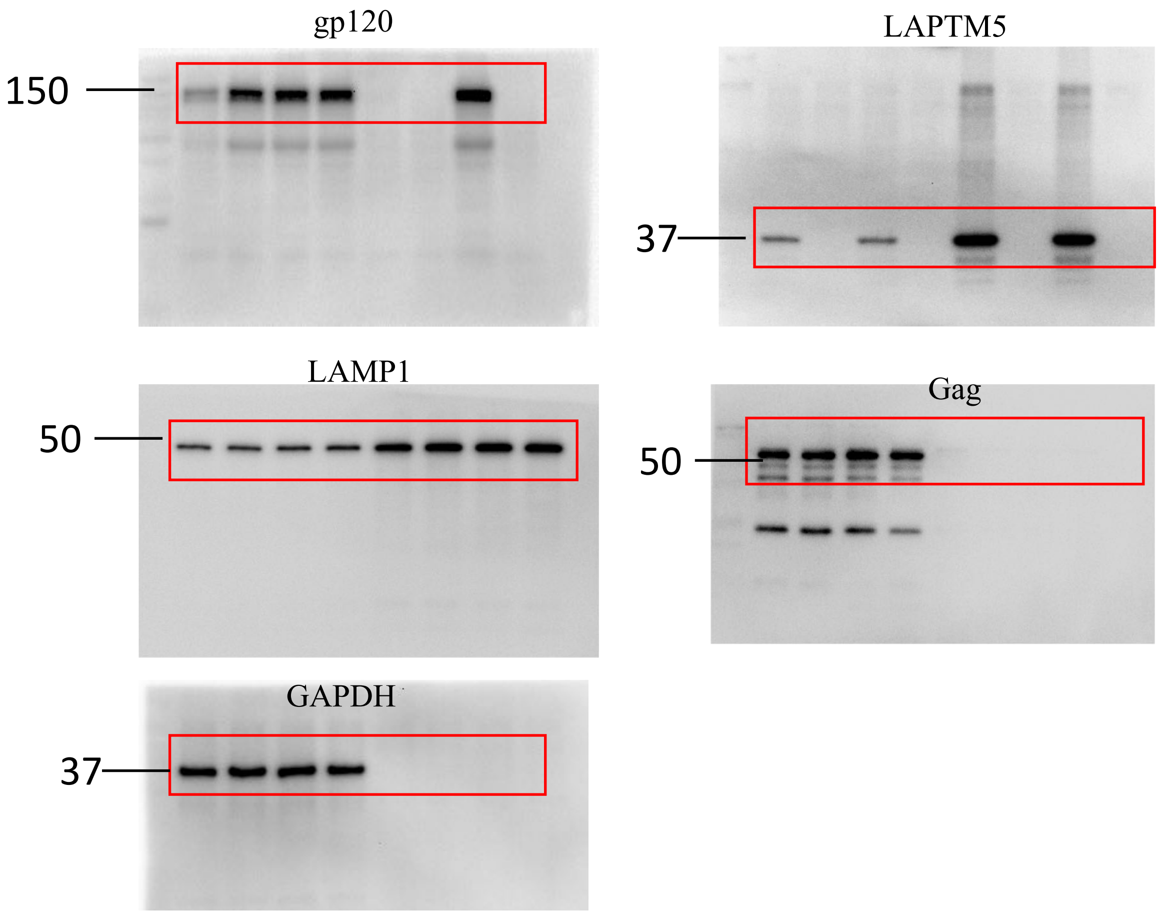
gp120
150
LAPTM5
37
LAMP1
50
Gag
50
GAPDH
37

Fig. 6a

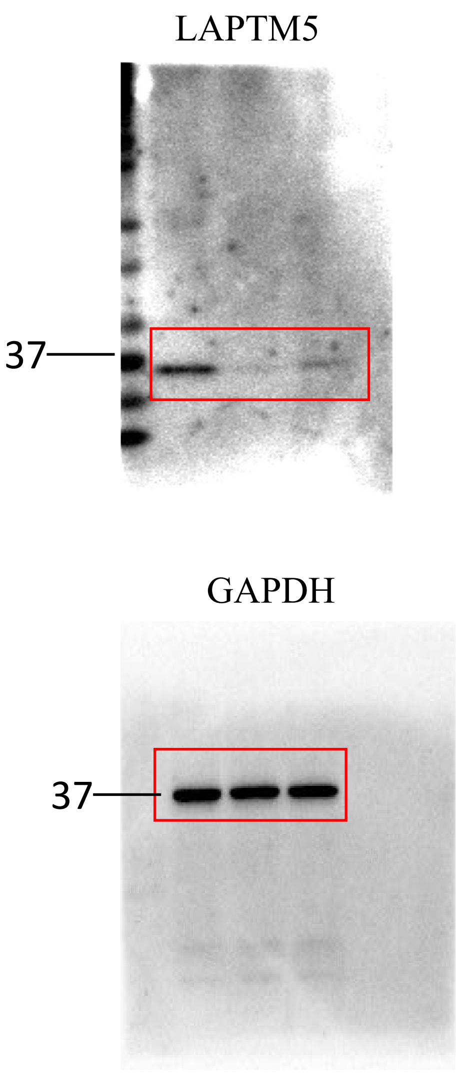
LAPTM5
37
GAPDH
37

Fig. 6d

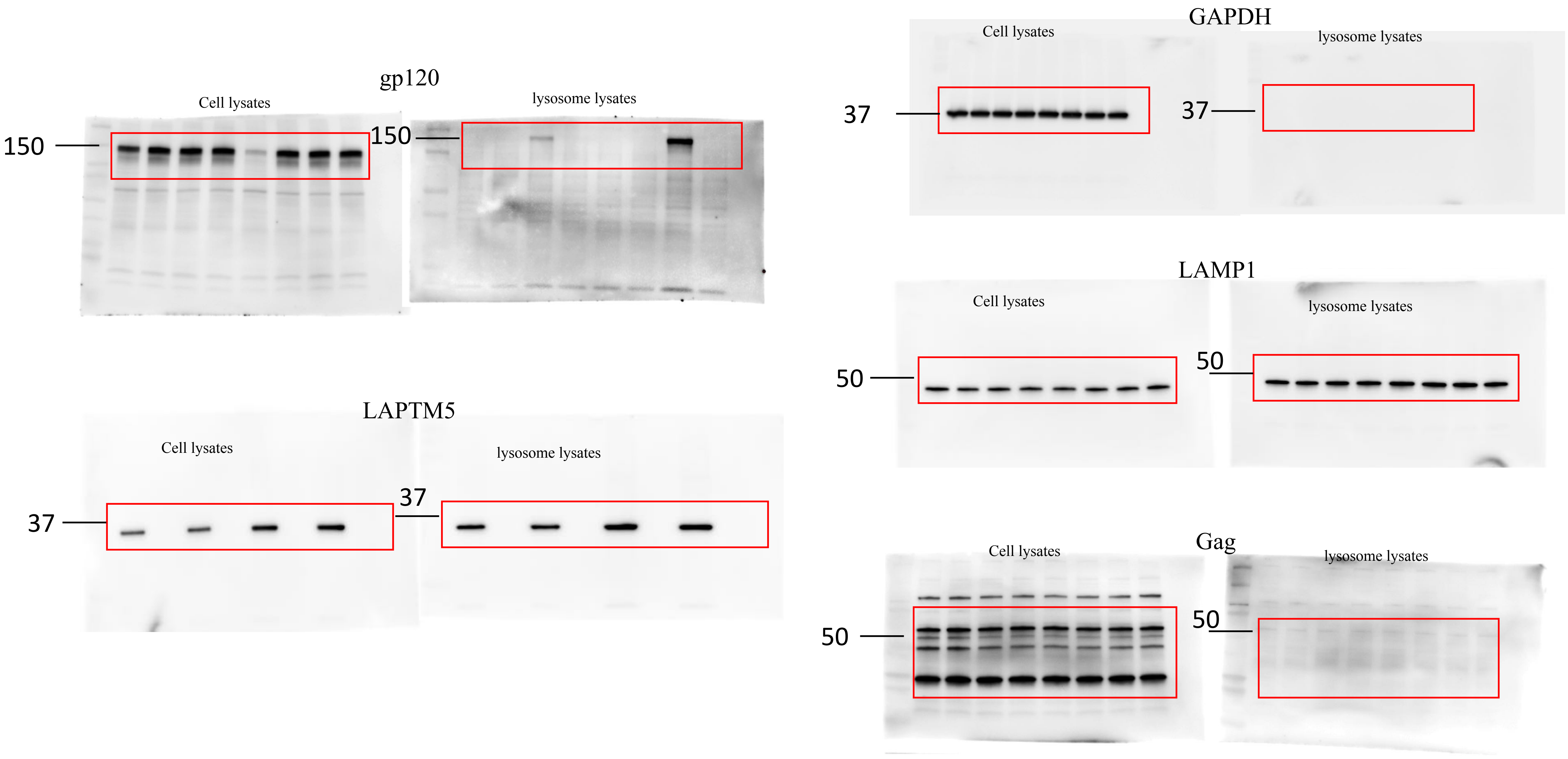
gp120
Cell lysates
lysosome lysates
150
150
LAPTM5
Cell lysates
lysosome lysates
37
37
GAPDH
Cell lysates
lysosome lysates
37
37
LAMP1
Cell lysates
lysosome lysates
50
50
Gag
Cell lysates
lysosome lysates
50
50

Fig. 7a

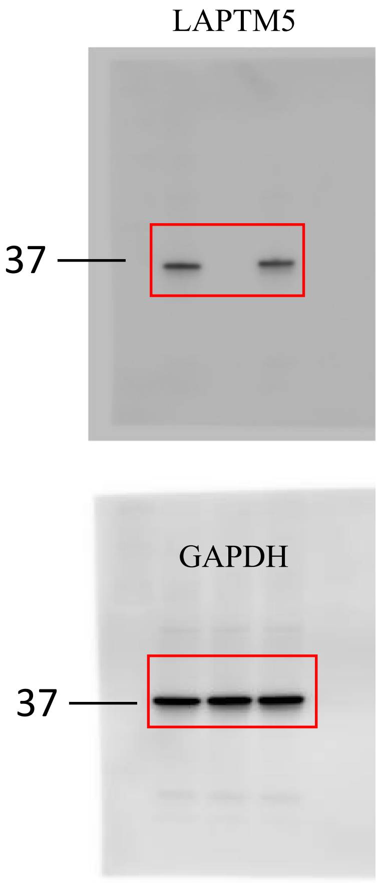

Fig. 7b

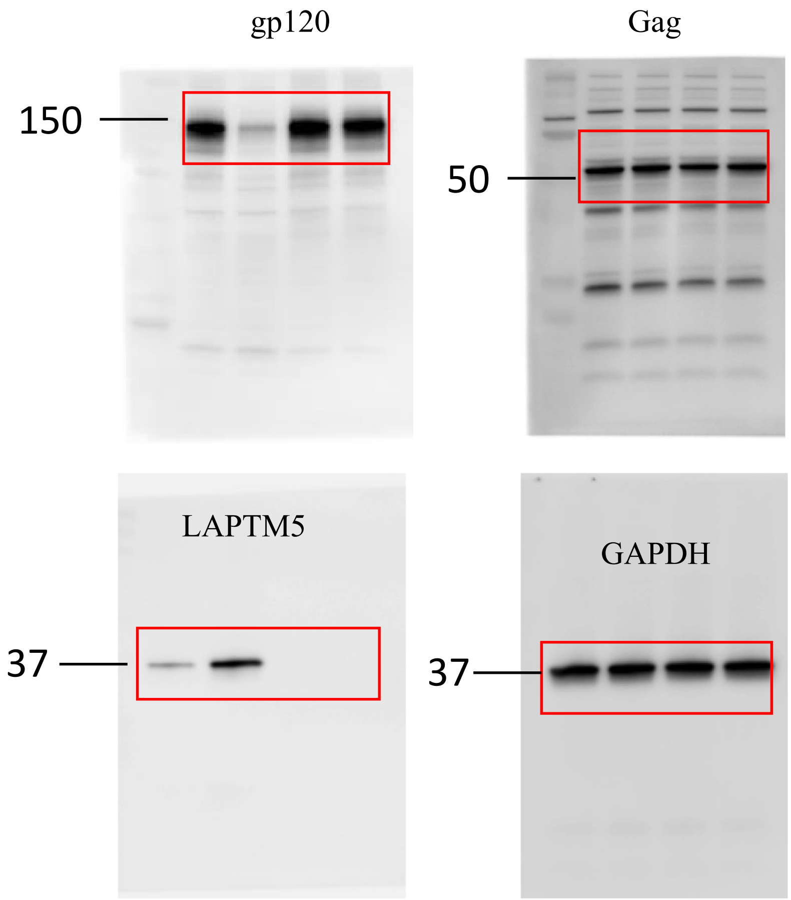

Fig. 7c

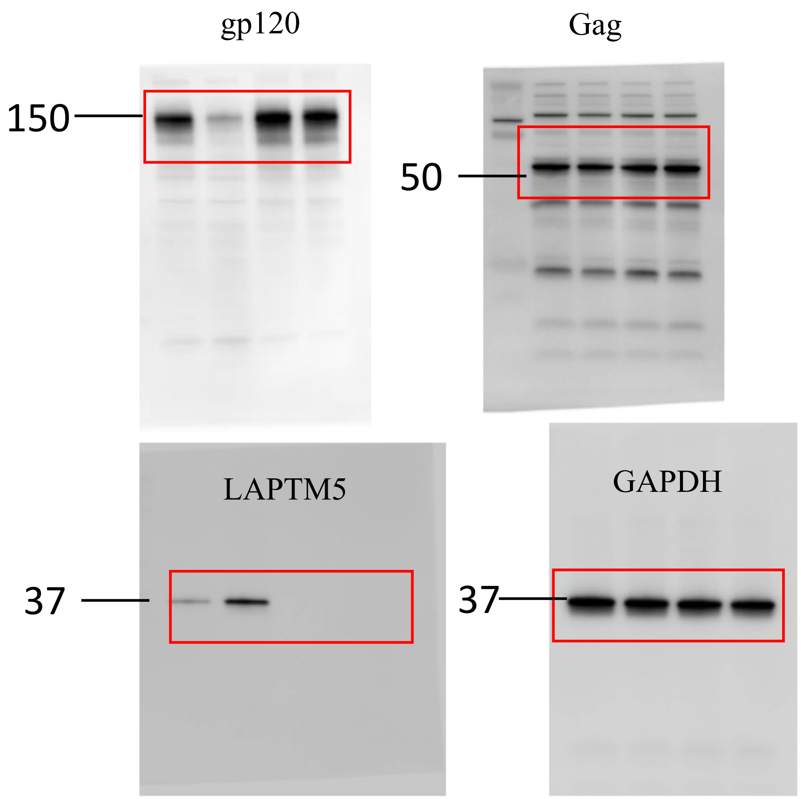

Fig. 7d

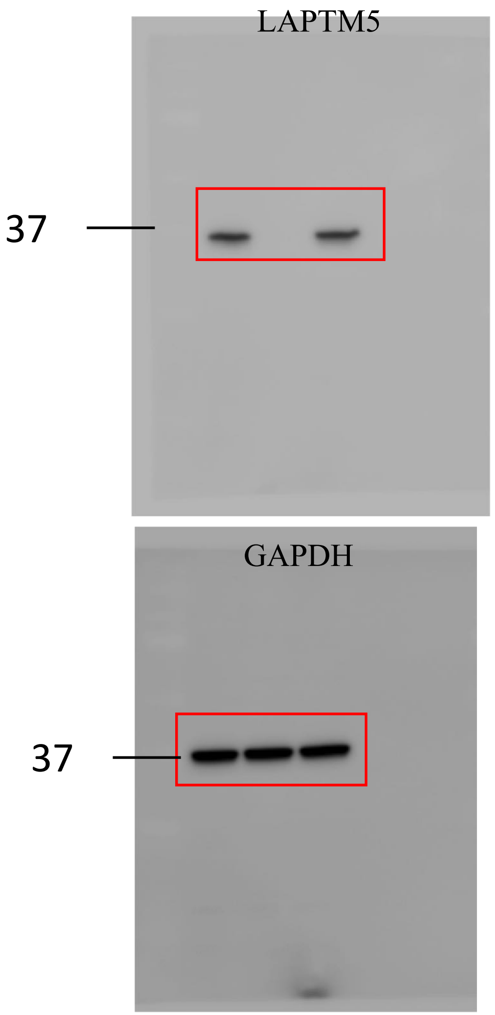

Supplementary Figure 1c

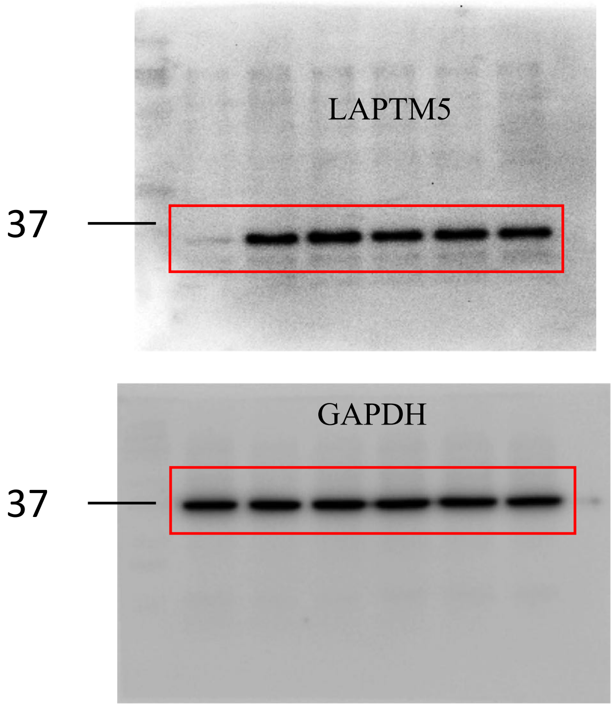

Supplementary Figure 1e

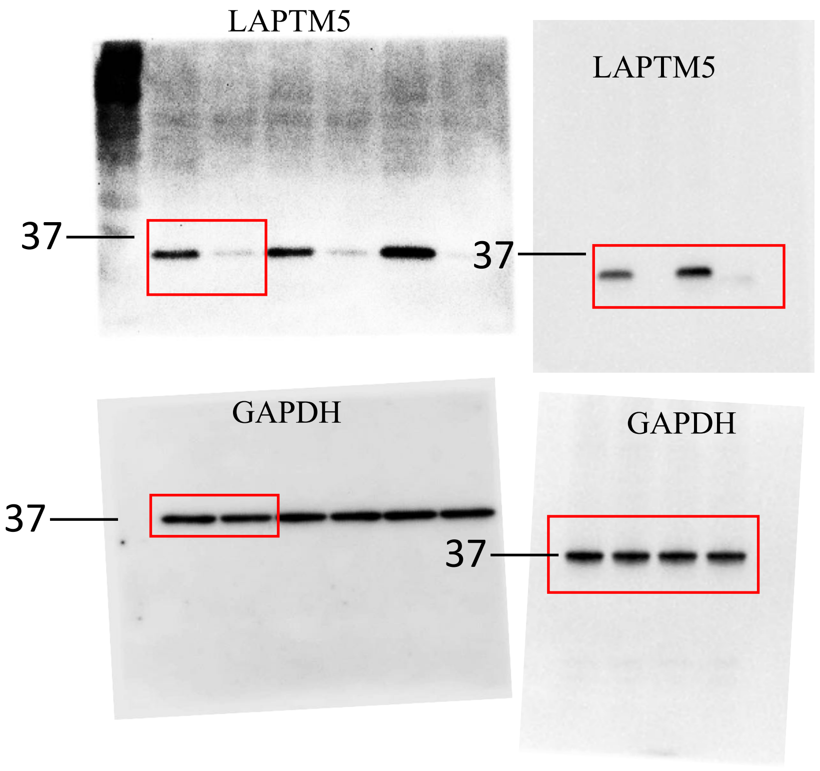

Supplementary Figure 1f

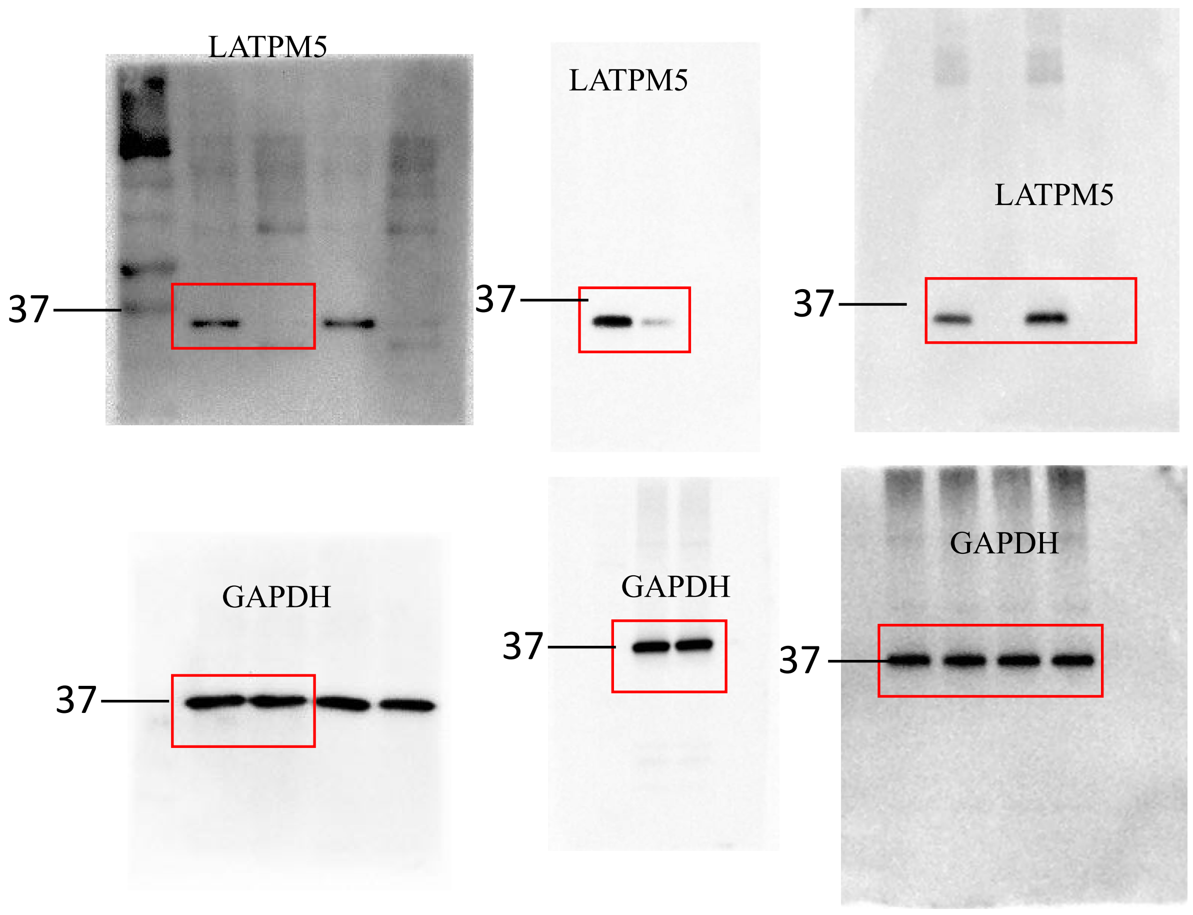

Supplementary Figure 2b

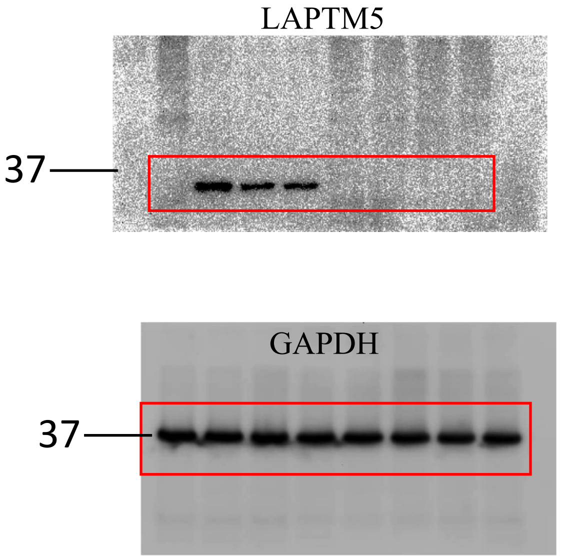

Supplementary Figure 2d

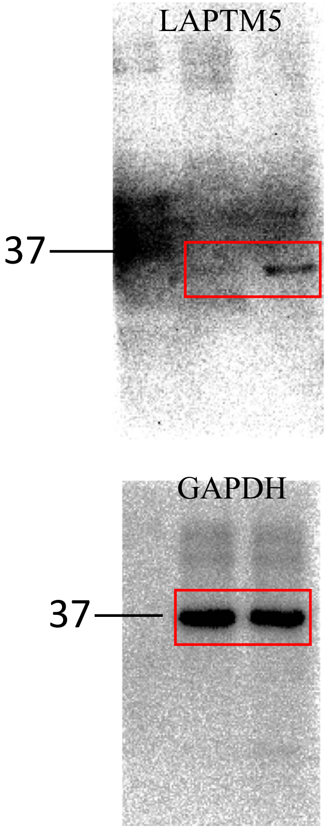

Supplementary Figure 3a

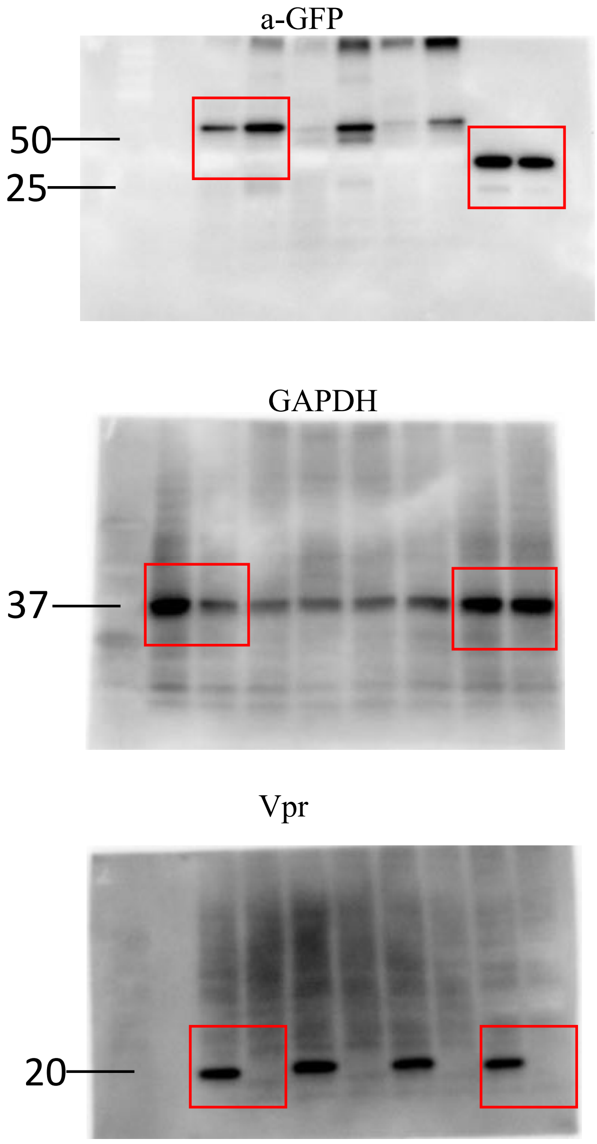

Supplementary Figure 3b

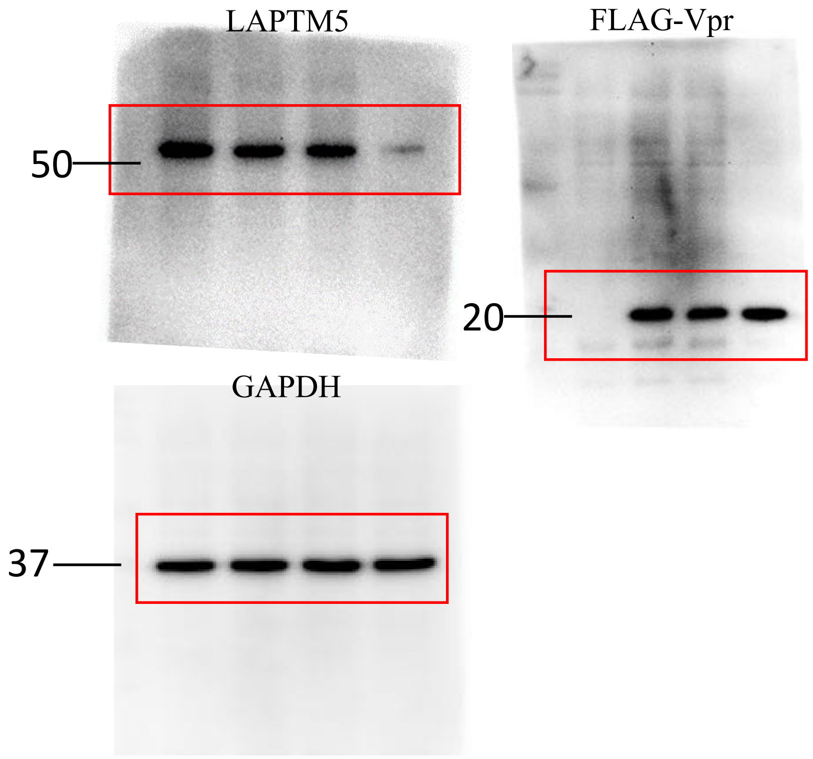

Supplementary Figure 3c

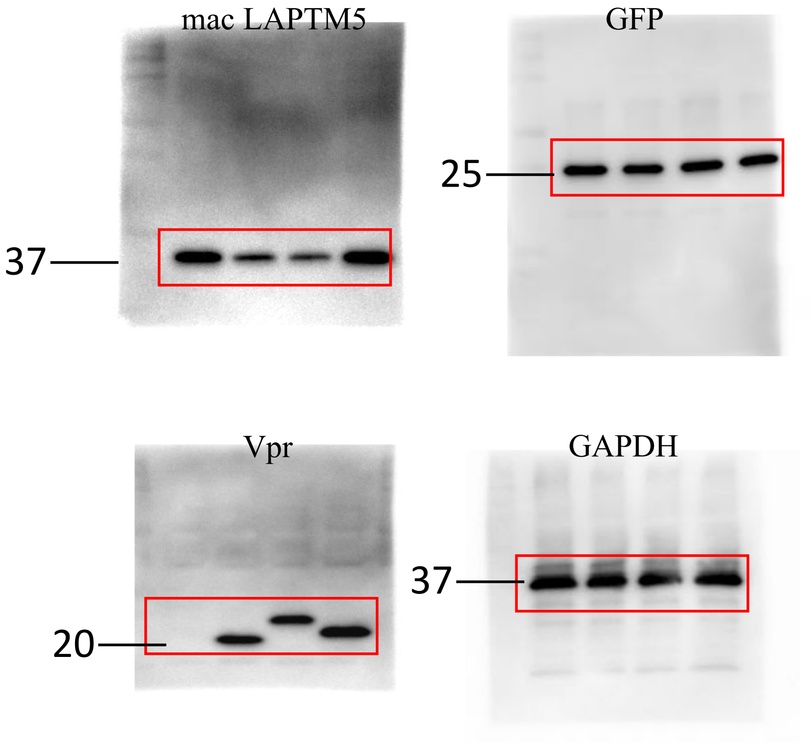

Supplementary Figure 3d

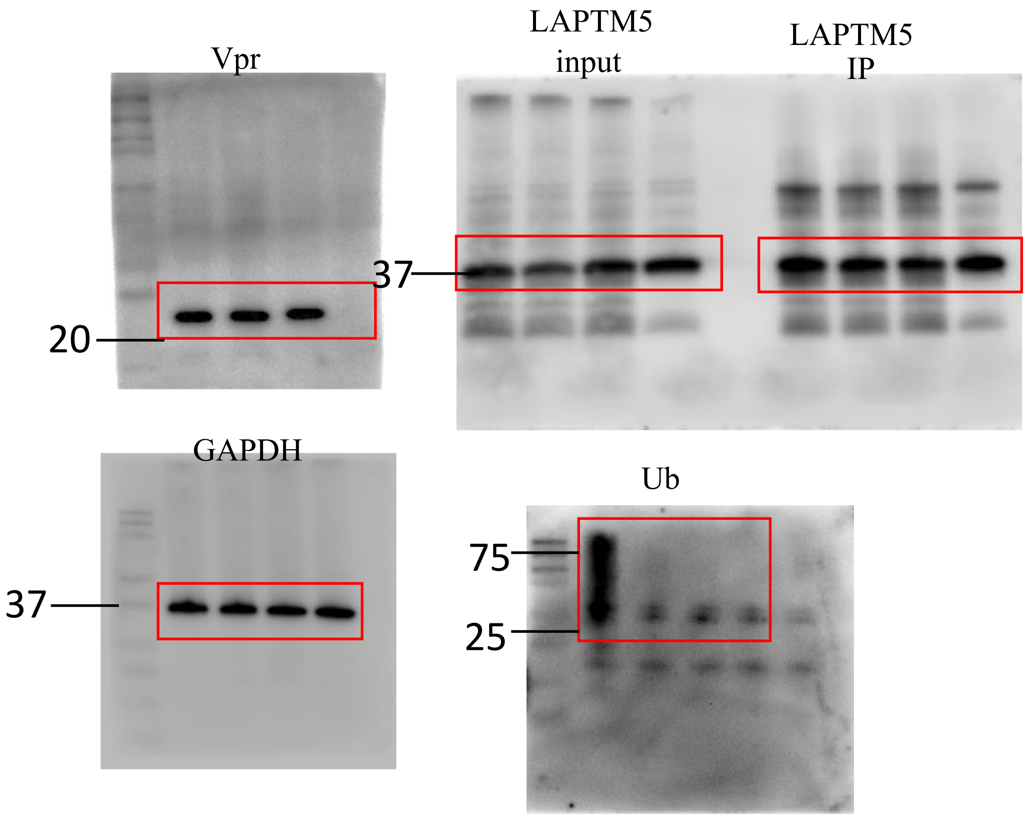

Supplementary Figure 4b

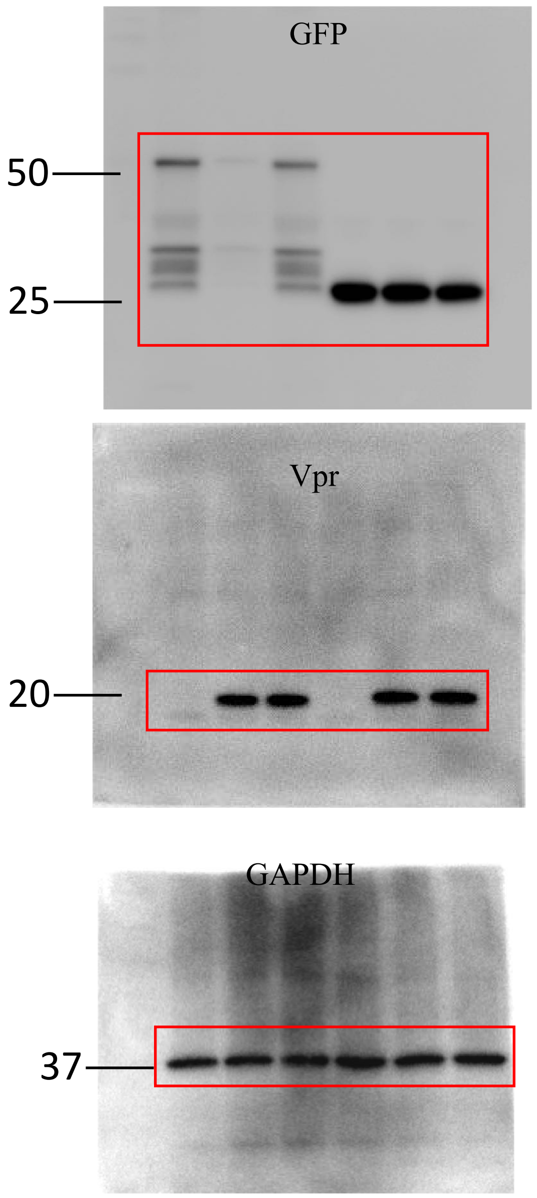

Supplementary Figure 4d

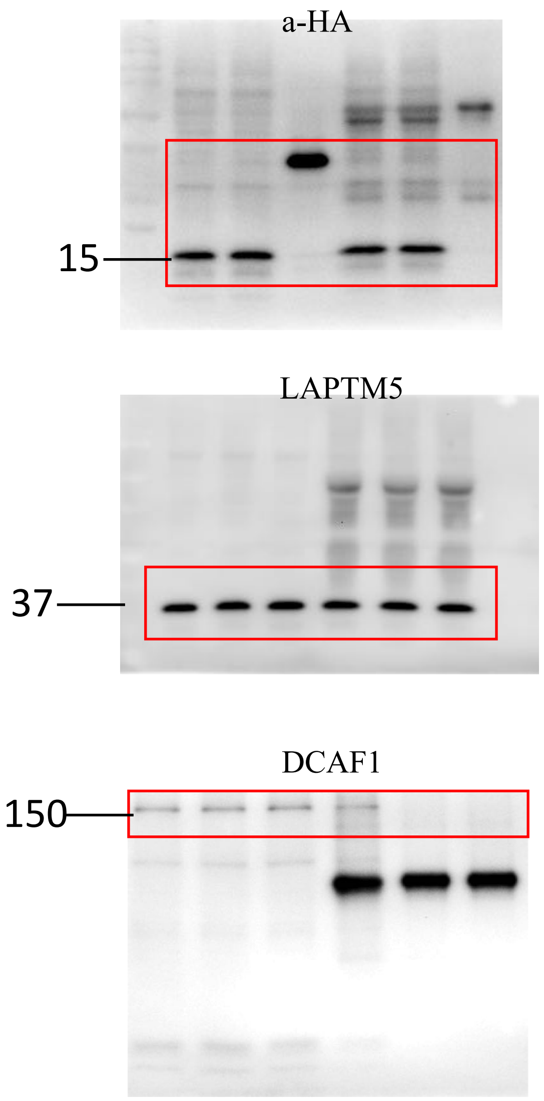

Supplementary Figure 5a

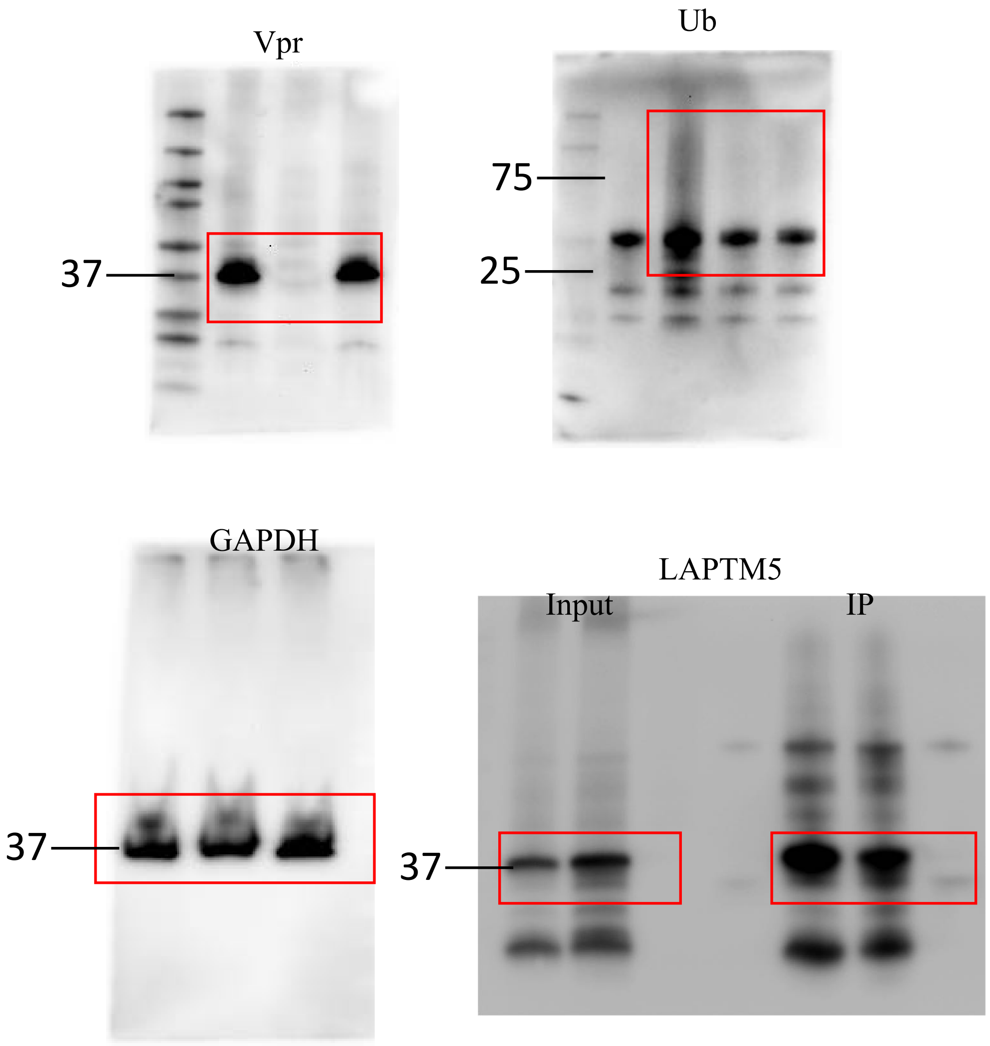

Supplementary Figure 5b

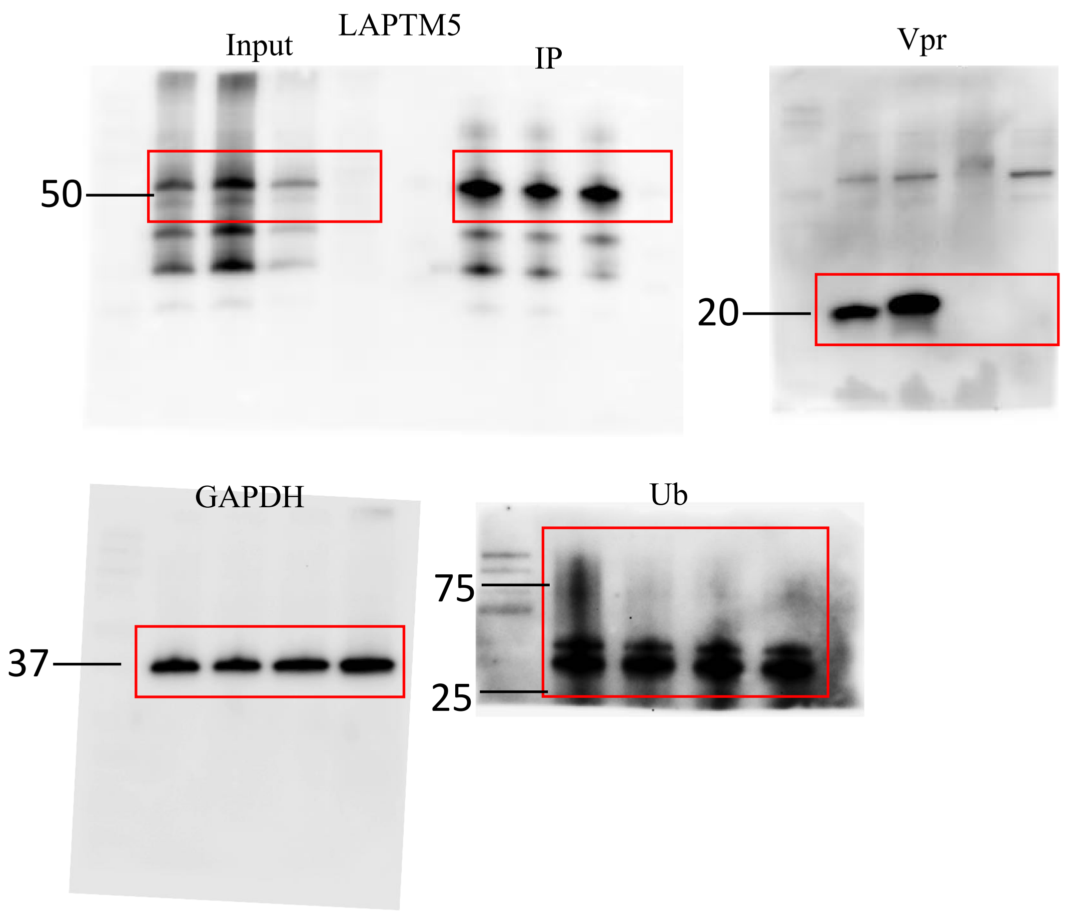

Supplementary Figure 5c

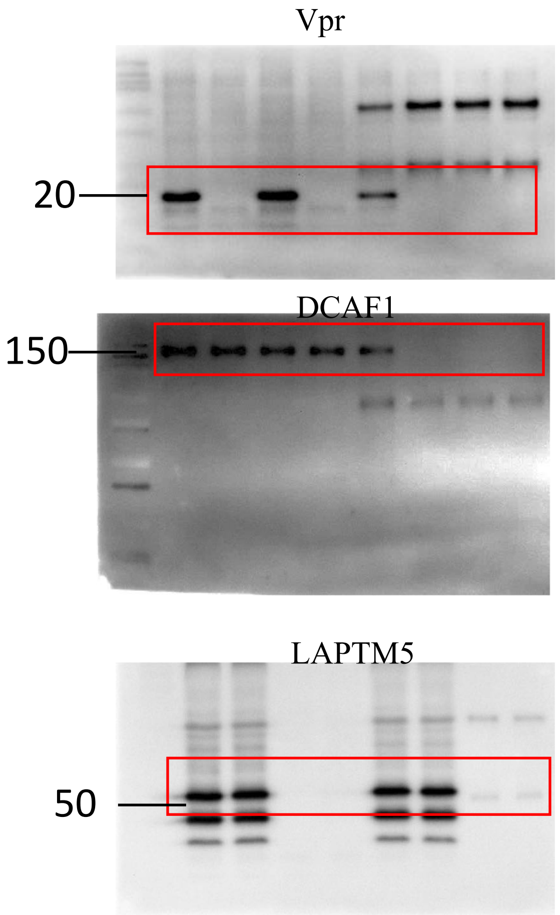

Supplementary Figure 5d

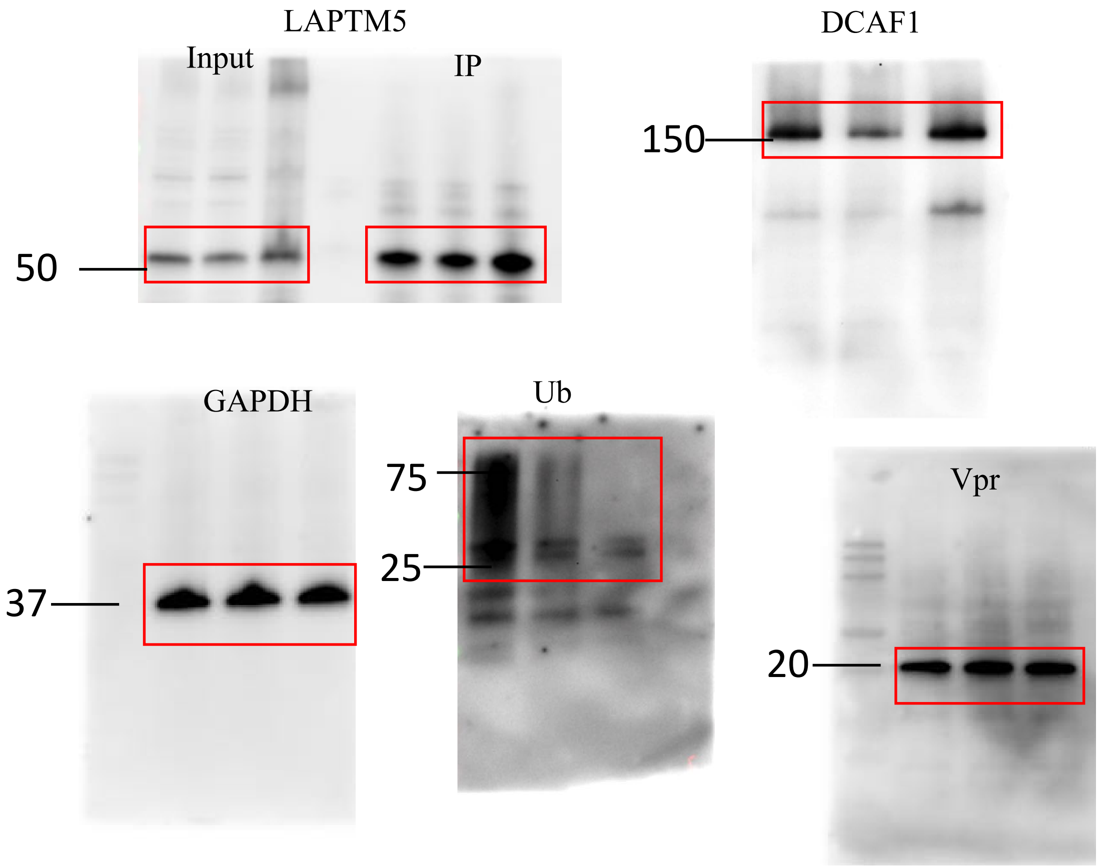

Supplementary Figure 6a

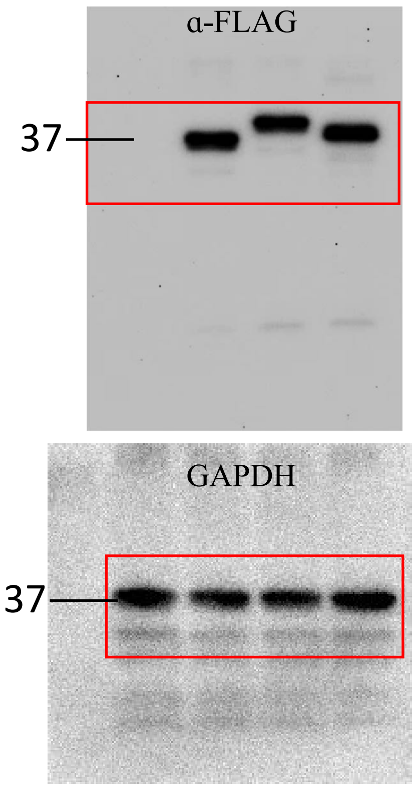

Supplementary Figure 6b

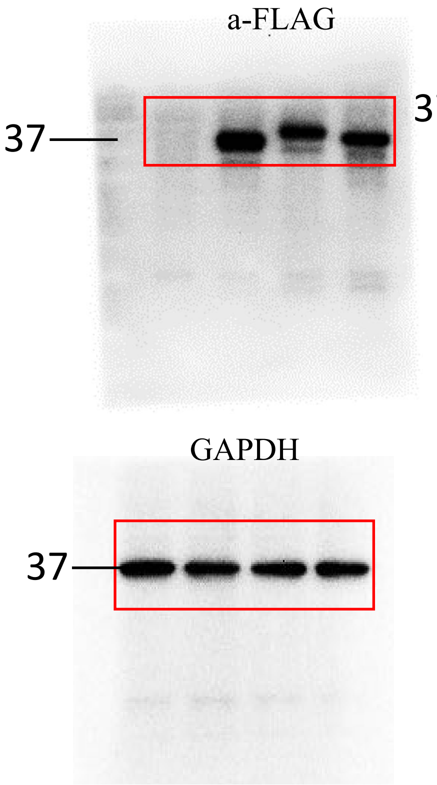

Supplementary Figure 6c

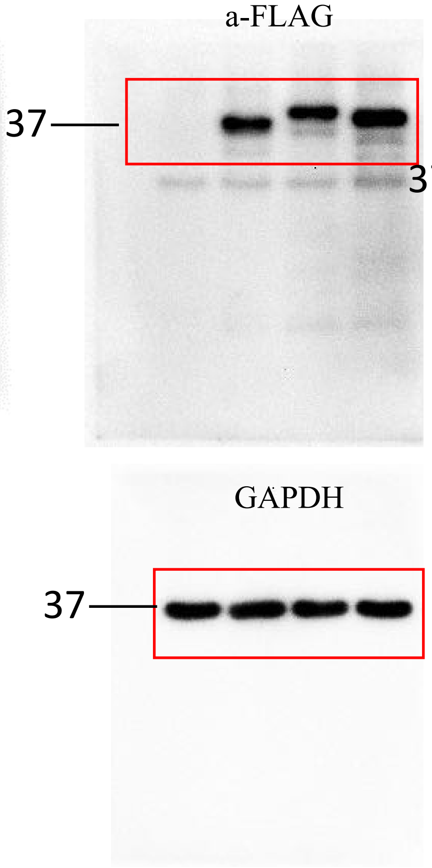

Supplementary Figure 6d

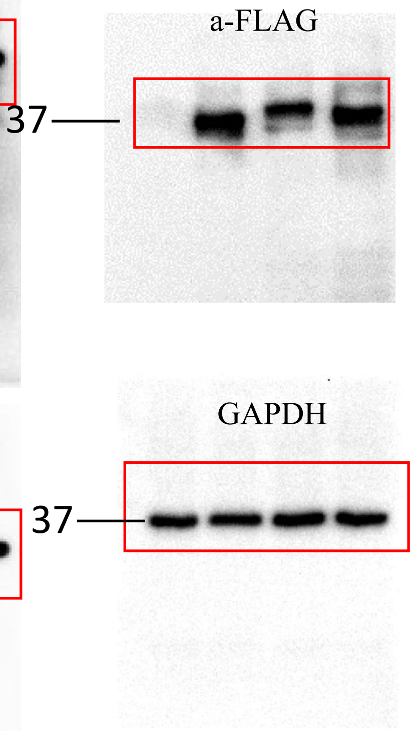

Supplementary Figure 6e

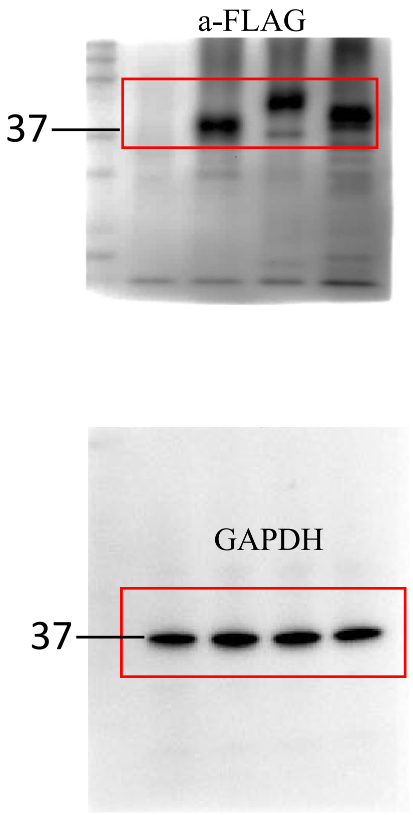

Supplementary Figure 6f

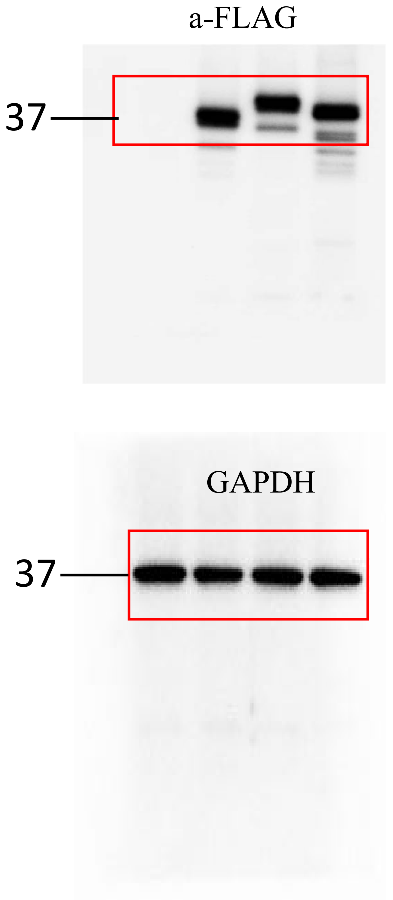

Supplement: Supplementary file 4 — Source Data [file 41467_2021_24087_MOESM4_ESM.zip › Source Data/Supplementary uncropped blots.pdf]
